# A systems-level atlas of carbon-response transcriptional states in *Escherichia coli*

**DOI:** 10.1073/pnas.2531884123

**Published:** 2026-07-01

**Authors:** Jongoh Shin, Arjun Patel, Xuwen A. Lou, Edward Alexander Catoiu, Jayanth Krishnan, Ying Hefner, Richard Szubin, Jaemin Sung, Hyeoncheol Francis Son, Daniel C. Zielinski, Bernhard Ørn Palsson

**Affiliations:** ^a^ Department of Bioengineering, University of California, San Diego, La Jolla, CA 92093; ^b^ Department of Biological Sciences, College of Natural Sciences, Chonnam National University, Gwangju 61186, Republic of Korea; ^c^ Institute of Synthetic Biology for Carbon Neutralization, Chonnam National University, Gwangju 61186, Republic of Korea; ^d^ School of Biological Sciences and Technology, College of Natural Sciences, Chonnam National University, Gwangju 61186, Republic of Korea; ^e^ Novo Nordisk Foundation Center for Biosustainability, Technical University of Denmark, Lyngby 2800, Denmark; ^f^ Department of Pediatrics, University of California, San Diego, La Jolla, CA 92093

**Keywords:** nutrient response, carbon catabolite repression, transcriptional regulatory networks, iModulon, systems biology

## Abstract

*Escherichia coli* encounters chemically diverse carbon sources, and the observed outputs of its transcriptional regulatory network (TRN) vary with substrate chemistry, metabolic entry route, and growth physiology. Here, we compiled PRECISE-NP881, an 881-condition transcriptome compendium comprising 346 RNA-seq profiles generated for this study during growth on 43 individual carbon sources, and used independent component analysis to quantify condition-specific activities of 137 iModulons, defined here as statistically independent gene-expression modules. We identified 25 carbon-catabolism iModulons and summarized their activity patterns across the 43 substrates into four activity-defined substrate groups. These activity patterns were associated with measured growth rates, substrate chemical classes, central-metabolic entry routes, carbon-normalized stoichiometric yield, and model-estimated proteome allocation. Faster-growing sugar conditions showed low CRP-linked iModulon activity, whereas slower-growing conditions showed elevated, condition-specific activity of CRP-linked and substrate-specific catabolic iModulons. TCA-entry and amino acid–associated conditions were linked with NtrC-1 and Propionate iModulon activities, with targeted knock-out assays supporting the conditional physiological relevance of selected propionyl-CoA-associated genes. A subset of nitrogen-containing, slower-growth conditions with predicted ammonium release induced the cryptic prophage-associated SgcABCEQX iModulon. Projection of an independent glucose starvation/refeeding time-course dataset revealed overlapping dynamics among selected carbon-catabolism iModulons and coordinated changes in growth- and stress-associated TRN outputs. Together, these results provide a systems-level atlas of observed carbon-responsive transcriptional states and systematize carbon physiology at scale.

## Significance

Microbial environments contain diverse carbon sources that impose different physiological demands. Using an *Escherichia coli* transcriptome compendium spanning 43 individual carbon substrates, together with growth phenotyping and metabolic modeling, we defined 25 carbon-catabolism gene-expression modules (iModulons) and grouped their activities into four substrate-response patterns. Preferred sugars showed limited transcriptional remodeling, whereas slower-growth substrates were associated with broader engagement of alternative catabolic and stress-linked modules. We also identified an NtrC and propionate-linked response associated with selected TCA-entry and amino acid–derived substrates, and a cryptic-prophage SgcABCEQX module induced under a subset of nitrogen-containing conditions. This atlas provides a quantitative reference for interpreting nutrient-responsive transcriptomes and generating hypotheses about carbon physiology in *E. coli*.

Microorganisms rarely encounter a single, saturating carbon substrate in nature. In the mammalian gut, soil, or surface waters, *Escherichia coli* confronts shifting mixtures of sugars, organic acids, amino acids, and nucleosides (1, 2). Classical carbon-catabolite repression (CCR) explains preferential glucose utilization and repression of alternative catabolic regulons: Abundant glucose depresses intracellular cyclic AMP, reduces cAMP–CRP activity, and supports high glycolytic flux (3, 4). When carbon conditions change, multiple global regulators—including cAMP–CRP, (p)ppGpp, and RpoS—contribute to transcriptional adaptation (5–8). However, most studies have focused either on individual substrates or on a small number of well-characterized transitions, leaving open how these responses are organized across a broader landscape of carbon environments.

Quantitative studies now show that carbon adaptation is not well captured by a simple on/off picture. Proteome-allocation studies show that broadening catabolic capacity consumes cellular protein resources (9, 10), and transcriptomic studies suggest that different regulatory programs can be engaged to different extents depending on nutrient context (4, 11). These observations motivate a systems-level description of carbon-responsive transcriptional states across many chemically distinct substrates.

A systems-level map of how these transcriptional regulatory network (TRN) outputs vary across diverse carbon substrates remains incomplete. Three questions are central. First, across chemically diverse carbon sources, how does carbon-responsive transcription decompose into modular CRP-linked and substrate-associated components? Second, which additional modules are associated with subsets of slower-growth or stress-associated carbon-source conditions, including potential overflow- or stress-related states? Third, how do global growth- and stress-associated TRN outputs provide physiological context for these carbon-response states?

To address these questions, we compiled PRECISE-NP881, an 881-condition transcriptome compendium spanning 43 individual carbon substrates, and analyzed it with independent component analysis (ICA). ICA resolves statistically independent gene-expression modules, referred to as iModulons, that provide a top–down modular description of TRN outputs. Focusing on carbon-catabolism iModulons, we identified four reproducible activity-defined substrate groups, ranging from preferred sugars with limited transcriptional remodeling to slower-growth substrates associated with broader engagement of CRP-linked and substrate-specific catabolic modules. We then integrated growth phenotyping, standardized genome-scale metabolic modeling, targeted mutants for selected modules, and reanalysis of an independent starvation/refeeding time-course dataset to connect these transcriptional states with physiological context. Finally, we quantified phylogroup-level conservation of representative iModulons to distinguish widely conserved modules from lineage-specific ones.

## Results

### Transcriptomic Decomposition Resolves Carbon-Response iModulons Across Diverse Carbon Substrates.

**Fig. 1.**
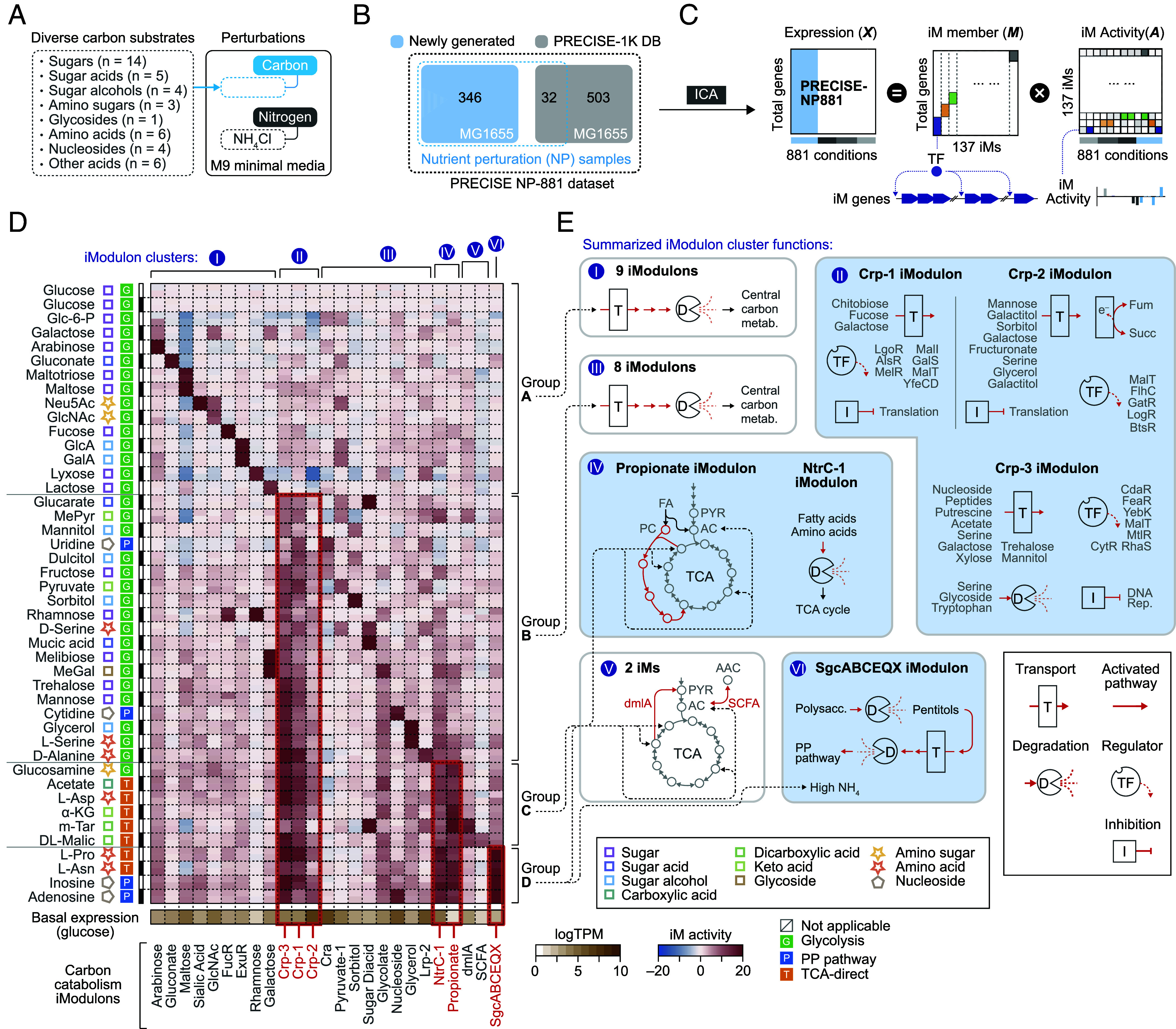
ICA of a nutrient-perturbation compendium resolves comprehensive carbon-responsive transcriptional modules in *E. coli*. (*A*) Carbon-perturbation design. Each of the 43 individual carbon substrates was added to M9 minimal medium to elicit nutrient responses. (*B*) Dataset composition. The PRECISE-NP881 compendium combines 346 generated RNA-seq profiles with 535 publicly available MG1655 profiles from the PRECISE-1 K dataset, yielding 881 growth conditions. (*C*) ICA workflow. ICA decomposed the 881-condition expression matrix (**X**) into 137 statistically independent gene-expression modules (“iModulons,” **M**) and their condition-specific activities (*A*). (*D*) Carbon-catabolism iModulons. Heat map of activities for 25 carbon-catabolism iModulons (columns) across the 43 carbon-source conditions (rows). Clustering of activity profiles identifies six iModulon clusters (I–VI); the utilization route and chemical class of each carbon substrate are indicated by the icons on the y-axis. Based on these activity patterns, carbon substrates fall into four activity-defined substrate groups (Group A–D, bar at bottom). The icon above each column denotes the central-metabolic entry route of the carbon substrate (green = glycolysis, blue = pentose-phosphate, orange = TCA-direct). (*E*) Functional synopsis of the six iModulon clusters. The schematic summarizes major gene functions and known regulatory associations represented by each cluster. Glc-6-P, D-Glucose-6-phosphate; Neu5Ac, N-Acetyl-neuraminic acid; GlcNAc, N-Acetyl-D-glucosamine; GlcA, D-Glucuronic acid; GalA, D-Galacturonic acid; MePyr, Methylpyruvate; MeGal, β-Methyl-D-galactoside; L-Asp, L-Aspartic acid; α-KG, α-Ketoglutaric acid; m-Tar, m-Tartaric acid; DL-Malic, D-Malic acid; L-Pro, L-Proline; L-Asn, L-Asparagine.

To build a condition-rich carbon transcriptome compendium, we assembled PRECISE-NP881 by profiling *E. coli* K-12 MG1655 growing in M9 medium supplemented with 43 individual carbon substrates spanning eight chemical classes (Fig. 1*A* and *SI Appendix*, Fig. S1*A* and Dataset S1). We merged 346 newly generated RNA-seq profiles with 535 public profiles from the PRECISE-1 K compendium (12), yielding 881 expression profiles with a median replicate Pearson *r* = 0.99 (Fig. 1*B* and *SI Appendix*, Fig. S1*B*). The complete sample-level manifest for PRECISE-NP881 is provided in Dataset S2, which lists all expression profiles and identifies the 346 RNA-seq profiles generated in this study. ICA resolved 137 iModulons, defined here as statistically independent gene-expression modules, that collectively accounted for 78% of total expression variance in the dataset (Fig. 1*C* and *SI Appendix*, Fig. S1 *C* and *D* and Dataset S2). 31 iModulons were enriched for carbon-metabolism genes (n = 444 genes in total). Of these, 25 showed substrate-dependent activity changes across the 43 individual carbon substrate conditions analyzed below (Dataset S2). Notably, 108 of the 444 genes (24%) are uncharacterized y-genes (13), underscoring that ICA associates poorly characterized genes with carbon-metabolism responses. Three of the 25 iModulons—CRP-3, dmlA, and SgcABCEQX—were absent from PRECISE-1K (12) and emerged only after incorporating the additional substrate conditions of PRECISE-NP881.

Clustering the activities of the 25 carbon-catabolism iModulons across the 43 individual carbon-source conditions organized the iModulons into six activity clusters (I–VI) and the substrates into four activity-defined substrate groups (A–D) (Fig. 1 *D* and *E*). Cluster I comprises nine iModulons that are active primarily on Group A substrates while cAMP–CRP activity remains low, highlighting CRP-independent modules associated with these faster-growing sugar conditions (Fig. 1 *D* and *E*). Cluster II contains three CRP-linked iModulons (Crp-1, Crp-2, and Crp-3) that are strongly induced across many Group B–D conditions (*SI Appendix*, Fig. S2 *A* and *B*); Crp-2 shows a more modest activity pattern than Crp-1 and Crp-3, consistent with promoter-context effects reported for CRP regulation (*SI Appendix*, Fig. S3) (12, 14). Each CRP-linked iModulon harbors a largely non-overlapping gene subset (*SI Appendix*, Fig. S2 *C*–*F*), indicating that ICA decomposed the broad CRP regulon into statistically separable, condition-associated components (14–16).

Cluster III includes eight substrate-specific catabolism iModulons—such as Glycerol, Sorbitol, Cra, and Glycolate iModulons—whose activities are observed in condition-specific combinations across Group B–D substrates. Cluster IV links an NtrC-1 iModulon—activating arginine, GABA, and fatty-acid catabolism—with a Propionate iModulon encoding the methylcitrate pathway (Fig. 1 *D* and *E*). Cluster V is restricted to a subset of Group C substrates. Cluster VI contains the prophage-derived *sgcABCEQX* operon and is observed in Group D conditions. Together, these six clusters provide a top–down organization of carbon-responsive TRN outputs across steady-state growth with individual carbon sources. We use this activity-based organization as a descriptive atlas for the quantitative analyses below.

### The Four Substrate Groups Show Distinct Growth Phenotypes and Modeling-Based Physiological Context.

**Fig. 2.**
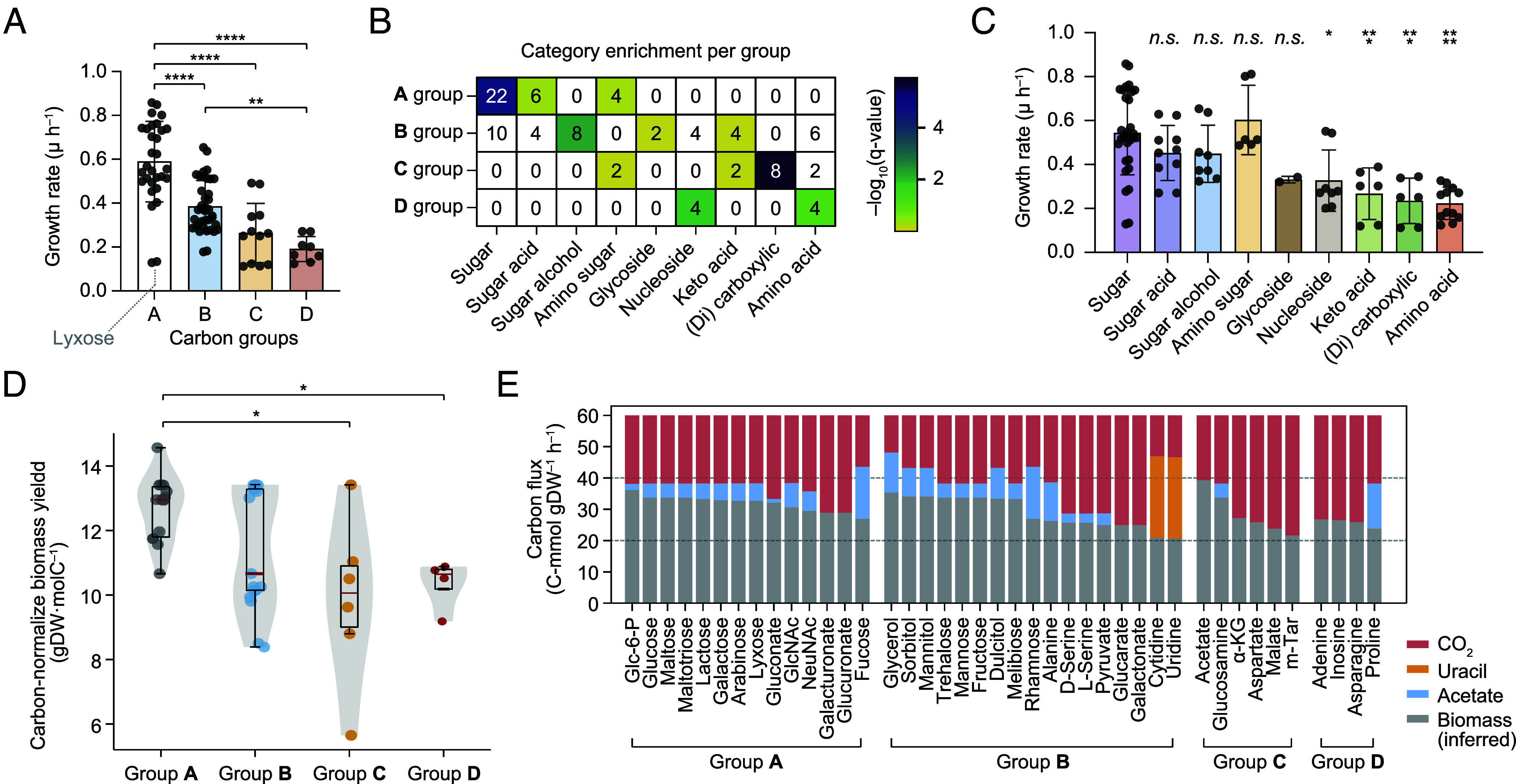
The four activity-defined substrate groups show distinct growth phenotypes and modeling-based physiological context. (*A*) Growth phenotypes. Comparison of average growth rates (μ h^−1^) for the four carbon substrate groups (A–D) defined in Fig. 1*D*. One-way ANOVA with Tukey’s post hoc test, **P < 0.01, ****P < 0.0001. (*B*) Enrichment of substrate chemical classes across groups. Heat map shows enrichment of nine compound classes (Fisher’s exact test, color indicates −log q-value); numbers give the count of carbon substrates per class. (*C*) Growth-rate distributions by compound class. Asterisks denote classes whose mean growth differs significantly from “Sugar” (Mann–Whitney test, ****P < 0.0001, ***P < 0.001, **P < 0.01, *P < 0.05, n.s. > 0.05). (*D*) Carbon-normalized maximum biomass yield predicted by flux balance analysis (FBA) across substrate groups. Substrate uptake bounds were scaled to enforce a fixed carbon influx (C_in = 60 C-mmol gDW^−1^ h^−1^; glucose-equivalent uptake 10 mmol gDW^−1^ h^−1^) with O_2_ LB = −20 mmol gDW^−1^ h^−1^. Violin plots show yield distributions across substrates in each group; points indicate individual substrates. Significances were assessed by Mann–Whitney *U* tests with Benjamini–Hochberg correction (*q < 0.05). (*E*) Carbon-fate partitioning predicted by FBA under standardized constraints. Bars sum to the fixed carbon influx (60 C-mmol gDW^−1^ h^−1^) and partition carbon into inferred biomass incorporation, fully oxidized CO_2_, and dominant exported metabolites under this constraint set (acetate and, for a subset of nucleoside conditions, uracil).

The four activity-defined substrate groups mirrored differences in substrate chemistry and central-metabolic entry routes (Fig. 2). Representative entry routes include glucose through glycolysis, α-ketoglutarate via the TCA cycle, and adenosine via the pentose-phosphate pathway (*SI Appendix*, Fig. S4). We therefore asked how the transcriptome-defined groups relate to measured growth phenotypes and standardized model-derived physiological summaries.

Experimentally measured growth rates differed markedly across groups (Fig. 2*A*). Group A substrates supported the highest growth rate (median 0.56 h^−1^) and were significantly faster than Groups B–D, whereas Group D exhibited the slowest growth (*P* < 0.003 for each successive comparison; Fig. 2*A*). Substrate chemistry tracked these phenotypes: Sugars and sugar acids dominated Groups A/B, whereas amino acids, organic acids, and nucleosides were enriched in Groups C/D (*q* < 0.014; Fig. 2 *B* and *C*).

To provide a standardized stoichiometric baseline for cross-substrate comparison, we performed genome-scale FBA under carbon-normalized uptake constraints. For each substrate, we scaled the substrate uptake lower bound to enforce a fixed carbon influx (60 C-mmol gDW^−1^ h^−1^; equivalent to a glucose uptake rate of 10 mmol gDW^−1^ h^−1^) and fixed the oxygen uptake bound at −20 mmol gDW^−1^ h^−1^. Under these standardized bounds, carbon-normalized maximum biomass yield differs across the activity-defined substrate groups (Fig. 2*D*), with Group A exhibiting higher yields than Group C/D and Group B spanning a wider range.

Using the same standardized constraints, we summarized how the fixed carbon influx was partitioned among inferred biomass incorporation, fully oxidized CO_2_, and the dominant exported metabolites under the model assumptions (Fig. 2*E*). Group A/B conditions generally supported higher inferred biomass incorporation, whereas Groups C/D showed increased carbon dissipation to CO_2_ under this constraint set; nucleoside/nucleobase substrates also exhibited prominent base-associated export (Fig. 2*E*). These analyses are used as standardized physiological context for the transcriptome-defined groups, not as direct predictions of in vivo uptake rates, overflow titers, or regulatory causality.

### NtrC-1 and Propionate iModulons Are Associated with Conditions Linked to Propionyl-CoA-Related Metabolic Stress.

**Fig. 3.**
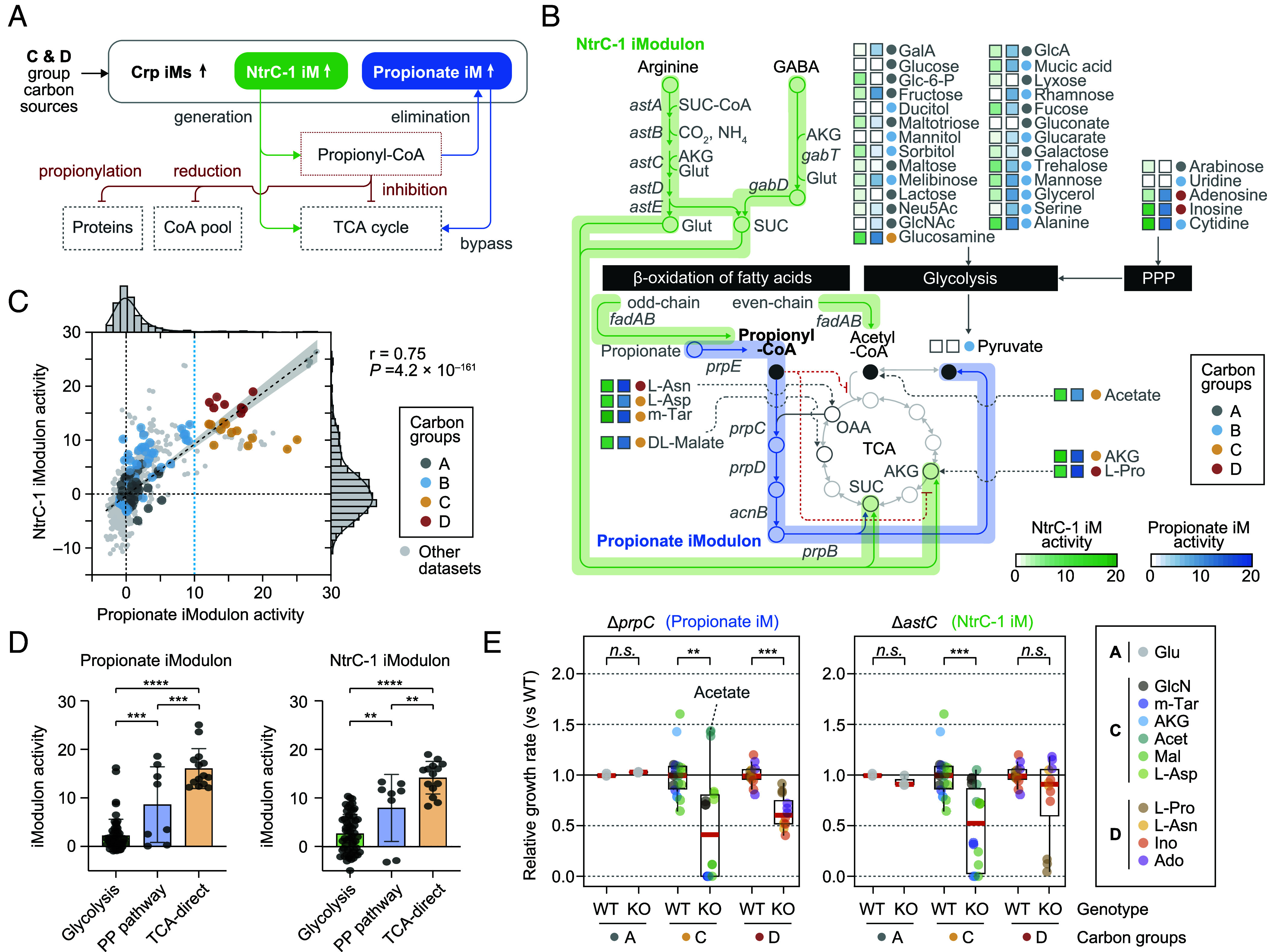
NtrC-1 and Propionate iModulons are associated with conditions linked to propionyl-CoA-related metabolic stress. (*A*) Conceptual summary of the relationship between NtrC-1 and Propionate iModulon activities and propionyl-CoA-associated metabolic context. (*B*) Metabolic pathway map showing representative genes in the NtrC-1 and Propionate iModulons and their connections to central metabolism. Genes belonging to NtrC-1 iModulon (green) and Propionate iModulon (blue) are mapped onto central metabolism. GalA, D-Galacturonic acid; Glc-6-P, D-Glucose-6-phosphate; Neu5Ac, N-Acetyl-neuraminic acid; GlcA, D-Glucuronic acid; L-Asn, L-Asparagine; L-Asp, L-Aspartic acid; m-Tar, m-Tartaric acid; AKG, α-Ketoglutaric acid; SUC-CoA, Succinyl-CoA; Glut, Glutamate; SUC, Succinate. (*C*) Scatterplot of Propionate iModulon vs. NtrC-1 iModulon activities across all 881 conditions (gray histograms on axes). Colored points highlight the 43 carbon-source experiments. (*D*) iModulon activities of Propionate and NtrC-1 iModulon by metabolic pathway type. One-way ANOVA with Tukey’s post hoc test, **P < 0.01, ***P < 0.001, ****P < 0.0001. (*E*) Relative growth of *ΔprpC* (propionate pathway) and *ΔastC* (arginine succinyl-transferase, NtrC-1 target) mutants compared with wild-type (WT) on representative Group A, C, and D carbon substrates. Welch *t* test (**P < 0.01, ***P < 0.001; n.s., not significant).

Growth on Group C/D substrates activates Cluster IV, which couples the NtrC-1 and Propionate iModulons with the global CRP response (Figs. 1*D* and 3*A*). Although NtrC is classically associated with nitrogen limitation (17), only one of the three NtrC iModulons—NtrC-1—was induced under ammonia-replete carbon-source conditions, consistent with prior reports linking ast-associated regulation to CRP/ArgR-dependent control (18). The NtrC-1 iModulon includes arginine-succinyl-transferase (*astABCDE*), GABA degradation (*gabTD*), and fatty-acid β-oxidation (*fadAB* + *fadJIE*), which can contribute succinate- and acetyl-CoA-linked inputs into central metabolism (Fig. 3*B* and *SI Appendix*, Figs. S5*A* and S6). These pathways can also be connected to propionyl-CoA generation, a metabolite known to inhibit pyruvate dehydrogenase and other TCA-cycle enzyme activities, sequester free CoA, and contribute to growth-retarding protein propionylation (19, 20). Unlike sugars that are first funneled through glycolysis, many Group C/D substrates enter central metabolism at or near the TCA cycle (*SI Appendix*, Fig. S4).

The Propionate iModulon (*prpBCDE*) encodes methylcitrate cycle enzymes that convert propionyl-CoA to succinate and pyruvate, a known route for propionyl-CoA conversion (19, 20) that can reduce propionyl-CoA-associated stress (Fig. 3*B* and *SI Appendix*, Fig. S5*B*). Across the 881 conditions, activities of the Propionate and NtrC-1 iModulons were strongly correlated (Pearson *r* = 0.75, *P* < 4 × 10^−181^) and positively associated with the three CRP-linked iModulons (Pearson *r* > 0.41, *P* < 5 × 10^−5^; *SI Appendix*, Fig. S5*C*). This coactivity was most pronounced in Group C/D substrates (Fig. 3*C*). Both Propionate and NtrC-1 iModulon activities were ≥ 2.7-fold higher on non-glycolytic, TCA-entry- associated (Group C/D) than on Group A/B sugar conditions (Fig. 3*D*). These associations are consistent with the known PrpR/CRP-linked regulation of the *prp* operon under carbon-limited contexts (21).

Targeted mutants supported the conditional physiological relevance of genes associated with these modules. Deletion of *prpC* (methylcitrate synthase) reduced growth to <0.63 × WT on every Group C/D substrate except acetate but did not affect glucose, supporting that methylcitrate-cycle–mediated propionyl-CoA processing supports a condition-specific requirement for prpC-associated function under these selected conditions (Fig. 3*E* and *SI Appendix*, Fig. S5*D*). Likewise, deleting *astC* impaired growth on several amino/organic acids, although the defect was less severe. In silico deletion of *prpC* or *astC* in the iML1515 model under the same carbon-normalized constraints did not reduce predicted growth (*SI Appendix,* Fig. S5*E*), indicating that the experimental defects likely reflect stress, inhibition, or regulatory/physiological effects not represented in steady-state stoichiometric FBA.

Cluster V, comprising the dmlA and SCFA iModulons, was specifically activated on a subset of these substrates (Fig. 1 *D* and *E*). The dmlA iModulon was uniquely induced by m-tartaric acid (*P* = 0.001), and both dmlA and SCFA iModulon activities increased during growth on DL-malic acid (*P* = 0.005 and 0.014, respectively), consistent with additional substrate-associated responses around central carbon metabolism. These condition-specific activities suggest additional substrate-associated responses around central carbon metabolism, but their mechanistic roles require direct biochemical testing.

Together, the NtrC-1, Propionate, dmlA, and SCFA iModulons identify a subset of carbon-source conditions in which propionyl-CoA-associated metabolism and related central-metabolic stress responses are transcriptionally apparent.

### The SgcABCEQX iModulon Is Induced Under Nitrogen-Containing, Slower-Growth Conditions.

**Fig. 4.**
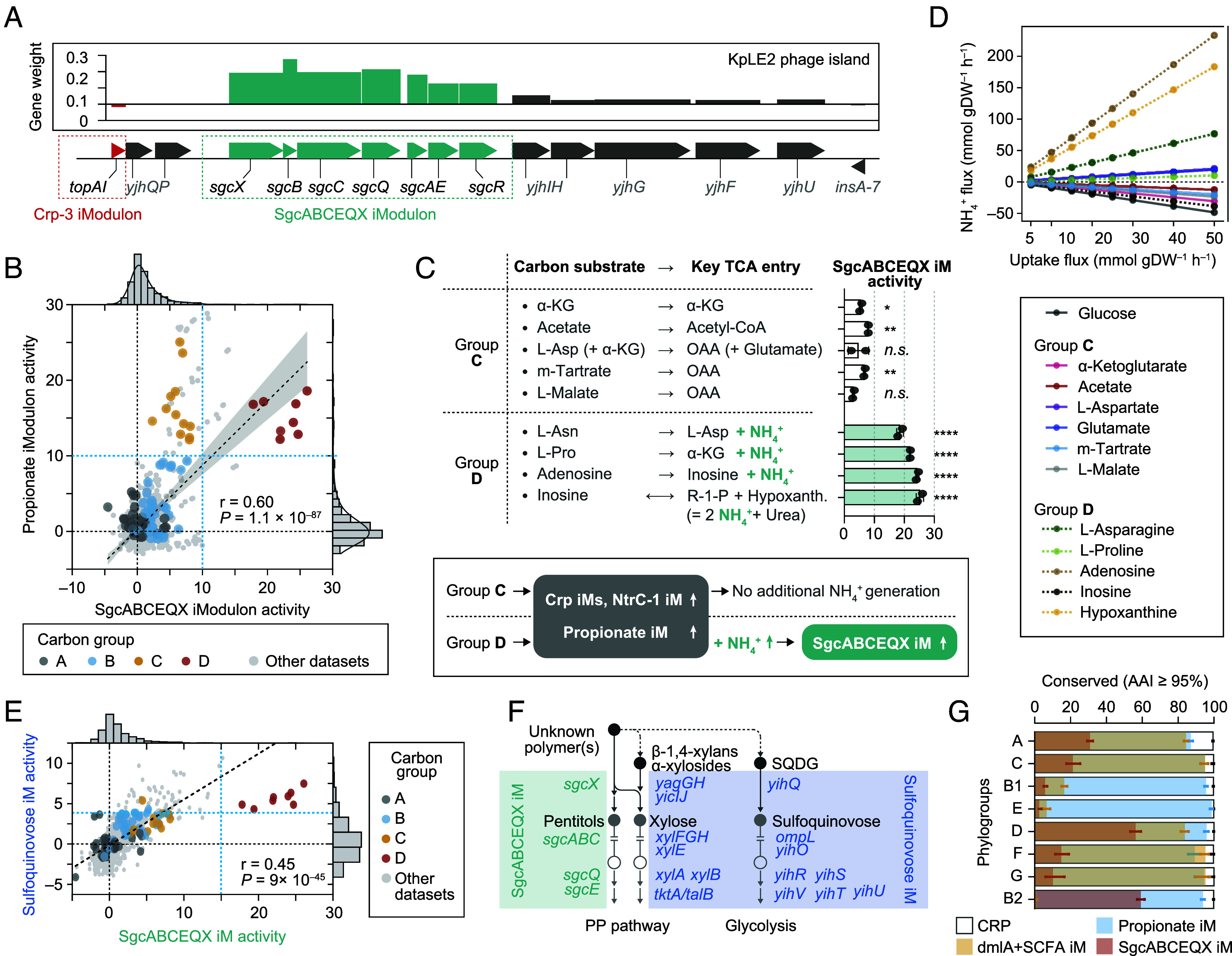
SgcABCEQX iModulon is induced under a subset of nitrogen-containing slower-growth conditions. (*A*) Gene-weight profile of the SgcABCEQX iModulon across the KpLE2 island. (*B*) Covariation of SgcABCEQX and Propionate iModulons. Activities of SgcABCEQX vs. Propionate iModulons in 881 transcriptomes (gray); the 43 carbon-source experiments are colored by defined carbon groups (A–D). (*C*) Ammonium release by Group D substrates. Predicted entry points of Group C and D substrates into the TCA cycle and the associated NH_4_^+^ released during conversion. Bar plots (means ± SD) show that SgcABCEQX activity is highest on Group D substrates that are predicted to liberate additional NH_4_^+^. Statistical significance was determined by one-way ANOVA with Dunnett’s post hoc test, comparing each carbon substrate to the glucose control. *P < 0.05, **P < 0.01, ***P < 0.001, ****P < 0.0001. ns, not significant. α-KG, α-Ketoglutaric acid; OAA, oxaloacetate; L-Asp, L-Aspartic acid; R-1-P, ribose-1-phosphate. (*D*) Flux-balance analysis of NH_4_^+^ export during substrate uptake. Solid lines, Group C substrates; dashed lines, Group D substrates. Inosine itself yields no net NH_4_^+^, but its intermediate hypoxanthine releases two NH_4_^+^ equivalents. (*E*) Positive correlation between the activities of the Sulfoquinovose and SgcABCEQX iModulons. (*F*) Proposed catabolic route linking the two iModulons. The SgcABCEQXiModulon (cyan) is shown as a putative working model for possible involvement in unusual carbohydrate or pentitol-related metabolism. The specific substrate and biochemical functions remain to be experimentally validated. The SulfoquinovoseiModulon (blue) channels sulfoquinovose from Sulfoquinovosyl diacylglycerols (SQDG) through *yihO*/PQRS genes into glycolysis, completing carbon utilization. (*G*) Phylogroup-level conservation of four gene sets in *E. coli* genomes. Horizontal bars show, for each Clermont phylogroup (23), the percentage of strains that harbor a conserved gene set, defined as amino acid identity (AAI) ≥ 95% computed from DIAMOND protein alignments to the MG1655 query set (24). A full-size version of this panel is provided in *SI Appendix*, Fig. S7*D*. Colors denote iModulon genes: CRP protein, Propionate iModulon, dmlA + SCFA iModulon, and SgcABCEQX iModulon. Error bars indicate 95% Wilson score intervals. Assemblies with ambiguous or cryptic phylogroup labels were excluded from the denominator. Mash distances to MG1655 were used to contextualize genome divergence (25).

Group D conditions strongly induced Cluster VI, the SgcABCEQX iModulon (hereafter, the Sgc iModulon; Fig. 4*A*). This iModulon comprises the six-gene *sgcABCEQX* operon embedded in the cryptic prophage island KpLE2; its functional annotation remains putative. Sequence homology and predicted protein-fold models suggest a possible connection to pentitol-related carbohydrate metabolism: SgcA/B/C resemble the IIA/IIB/IIC subunits of a phosphotransferase system (PTS) permease, SgcE resembles a ribulose-5-phosphate epimerase, SgcQ carries PTS chaperone-like features, and SgcX bears a zinc-dependent hydrolase signature that could cleave extracellular carbohydrate substrates (Dataset S3) (22). The specific substrate(s) and biochemical functions of this operon remain to be established.

Across PRECISE-NP881, Sgc iModulon activity covaried with the Propionate iModulon (Pearson *r* = 0.60, *P* = 1.1 × 10^−87^; Fig. 4*B*) but remained near baseline on most Group C substrates (median activity < 5; *P* > 0.05), while rising sharply 16 to 25-fold in Group D conditions (Fig. 4*C*). These conditions are enriched for nitrogen-containing compounds whose conversion to central-metabolic intermediates is predicted to release ammonium equivalents. Consistent with this, FBA under standardized aerobic bounds predicted higher net NH_4_^+^ generation potential for Group D substrates relative to Group C (Fig. 4 *C* and *D*). Thus, strong Sgc iModulon induction is associated with low-carbon-quality, nitrogen-containing carbon-source conditions with predicted ammonium release, rather than with carbon limitation alone.

Despite its strong induction signature, the Sgc iModulon was not required for growth under the laboratory conditions tested. Across standard laboratory substrates and 190 carbon sources in Biolog PM panels, Δ*sgc* mutants showed no growth defect relative to the parental strain (*SI Appendix*, Fig. S7 *A*–*C*). These results support the interpretation that the Sgc iModulon is a condition-specific transcriptional readout in our dataset, rather than a module required for the assayed individual-carbon-source growth phenotypes.

Within PRECISE-NP881, the Sgc iModulon showed its strongest transcriptional covariation with the Sulfoquinovose iModulon (Pearson *r* = 0.45, *P* = 9 × 10^−45^; Fig. 4*E*), which links the *yih* sulfoglycolysis operon with *xyl* operons converting D-xylose and xylosides to xylulose-5-phosphate. Based on this covariation and the putative annotations of *sgcABCEQX*, we propose a working model in which Sgc-associated functions could connect unusual carbohydrate or pentitol chemistry to pentose-phosphate-linked metabolism (Fig. 4*F*) (26). This model is presented as a hypothesis that will require biochemical validation.

Finally, to evaluate conservation of representative module-associated gene sets across *E. coli* lineages, we quantified average AAI to MG1655 ortholog sets across *E. coli* phylogroups and compared these values to whole-genome Mash distance (Fig. 4*G* and *SI Appendix*, Fig. S7*D*) (25, 27). CRP and the Propionate iModulon gene sets were broadly conserved across phylogroups (99.79 ± 0.18% and 93.74 ± 3.75%, respectively), whereas dmlA + SCFA iModulon genes showed marked phylogroup-specific loss (B1, E, B2; median 6.67 %), and *sgcABCEQX* was detected primarily in A, D, and a subset of B2 genomes [1,032/3,343 (30.87%), 600/1,066 (56.29%), and 1,279/2,160 (59.21%), respectively]. These patterns indicate that core carbon-response regulators and selected condition-associated modules differ in their phylogroup-level conservation.

### Global Growth- and Stress-Associated iModulons Provide Physiological Context for the Carbon-Response Groups.

Large-scale transcriptome analyses have highlighted an inverse relationship between growth-related functions and stress-associated transcriptional programs (28), consistent with proteome allocation constraints. We therefore asked whether the noncatabolic iModulons in PRECISE-NP881 provide a physiological context for the four activity-defined substrate groups (A–D).

**Fig. 5.**
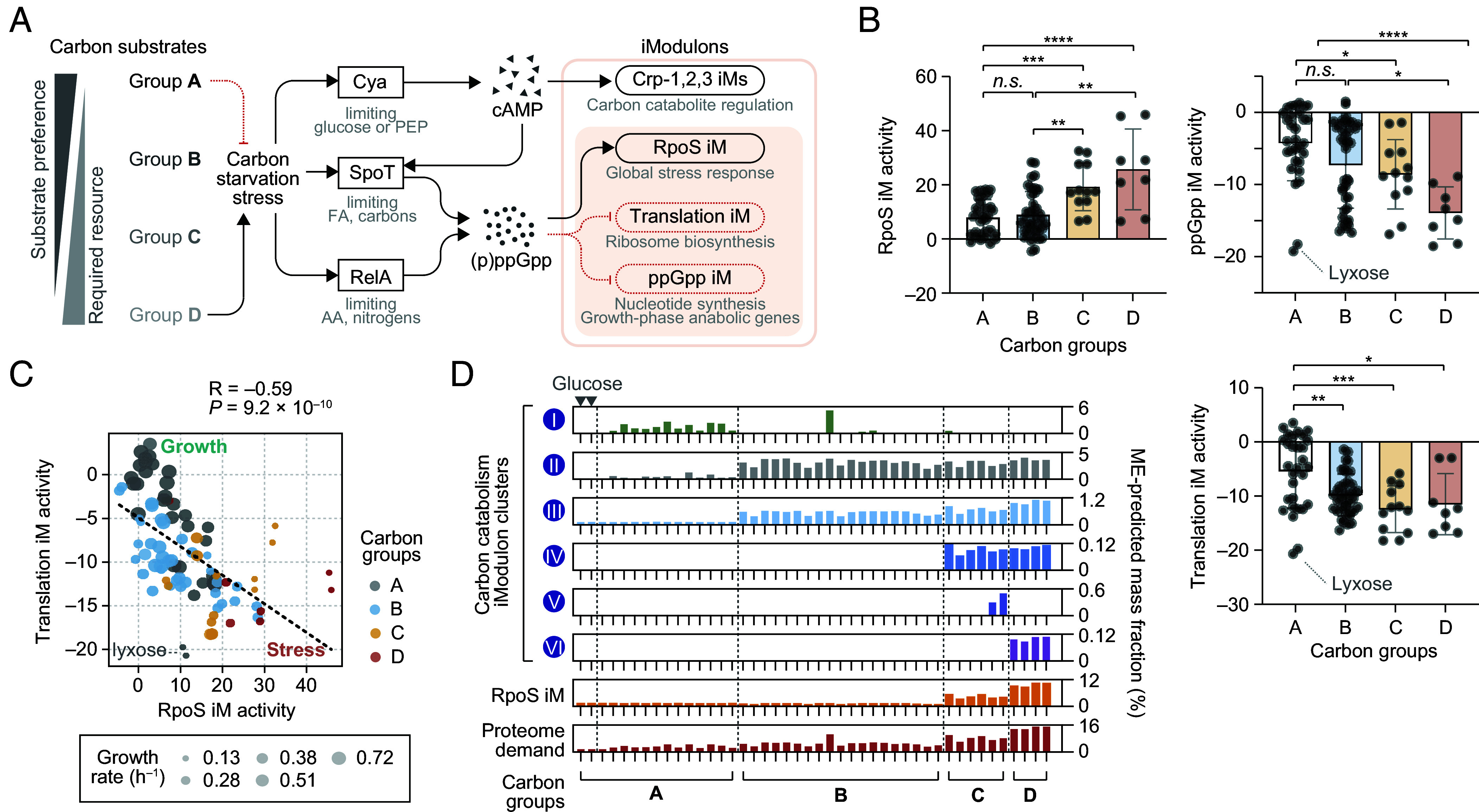
Global growth- and stress-associated iModulons provide physiological context for the carbon-response groups. (*A*) Conceptual summary of how activity-defined substrate groups relate to known growth- and stress-associated physiology. (*B*) Activities of RpoS, Translation, and ppGpp iModulons across substrate groups. One-way ANOVA with Tukey’s post hoc test, *P < 0.05, **P < 0.01, ***P < 0.001, ****P < 0.0001; n.s., not significant. (*C*) Relationship between Translation and RpoS iModulon activities across the 43 individual carbon-source conditions; symbol size indicates measured growth rate. (*D*) Genome-scale metabolism and expression (ME)-model-predicted proteome allocation for each of the 43 carbon substrates. For each of the 43 carbon substrates, bars show the mass fraction assigned to the six carbon-catabolism iModulon groups I–VI and to the RpoS stress iModulon (yellow). The bottom row, “Proteome demand,” aggregates the mass fractions of rows I–VI plus RpoS, representing the model-estimated mass fraction assigned to the indicated carbon-catabolism and stress-associated sectors under the simulation constraints.

Activities of global growth- and stress-associated iModulons varied across the four substrate groups (Fig. 5*A*). RpoS activity increased >2.3-fold in Groups C/D relative to Group A (*P* < 8 × 10^−4^; Fig. 5*B*), whereas Translation and ppGpp iModulon activities declined (<0.8-fold, *P* < 0.02) (*SI Appendix*, Fig. S8*A*). Here, the ppGpp iModulon corresponds to genes that are repressed under elevated (p)ppGpp; thus, lower activity is consistent with increased stringent-response signaling. Consistent with established growth-stress physiology, Translation iModulon activity decreased as RpoS activity increased across the 43 individual carbon conditions (Pearson *r* = −0.59, *P* = 9.2 × 10^−10^; Fig. 5*C*).

To place these transcriptional patterns in a quantitative allocation framework, we used ME-model simulations (29) to compute proteome fractions assigned to carbon-catabolism module sets (Clusters I–VI) and to the RpoS-associated regulons under the same substrate conditions (Fig. 5*D* and *SI Appendix*, Fig. S8*B*). The CRP-linked proteome accounted for ~5% of the proteome from Group B onward. For Group C/D substrates, the ME-model additionally allocated larger fractions to RpoS-associated stress functions, bringing the total estimated investment in carbon-catabolism-associated and stress-associated sectors to as much as approximately 16% in selected Group D substrates. Although specific substrates elicited idiosyncratic responses (e.g., OxyR iModulon induction on lyxose; *SI Appendix*, Fig. S8*C*), these local deviations did not alter the overall group-level pattern.

Collectively, these results connect the activity-defined substrate groups to known growth/stress physiology and model-estimated proteome requirements. Slower-growth carbon-source conditions were associated with lower Translation iModulon activity, higher RpoS iModulon activity, and larger model-estimated allocation to carbon-utilization and stress-associated sectors. These analyses contextualize the carbon-response atlas.

### An Independent Starvation/Refeeding Time Course Shows Overlapping Activation of Selected Carbon- and Stress-Associated iModulons.

**Fig. 6.**
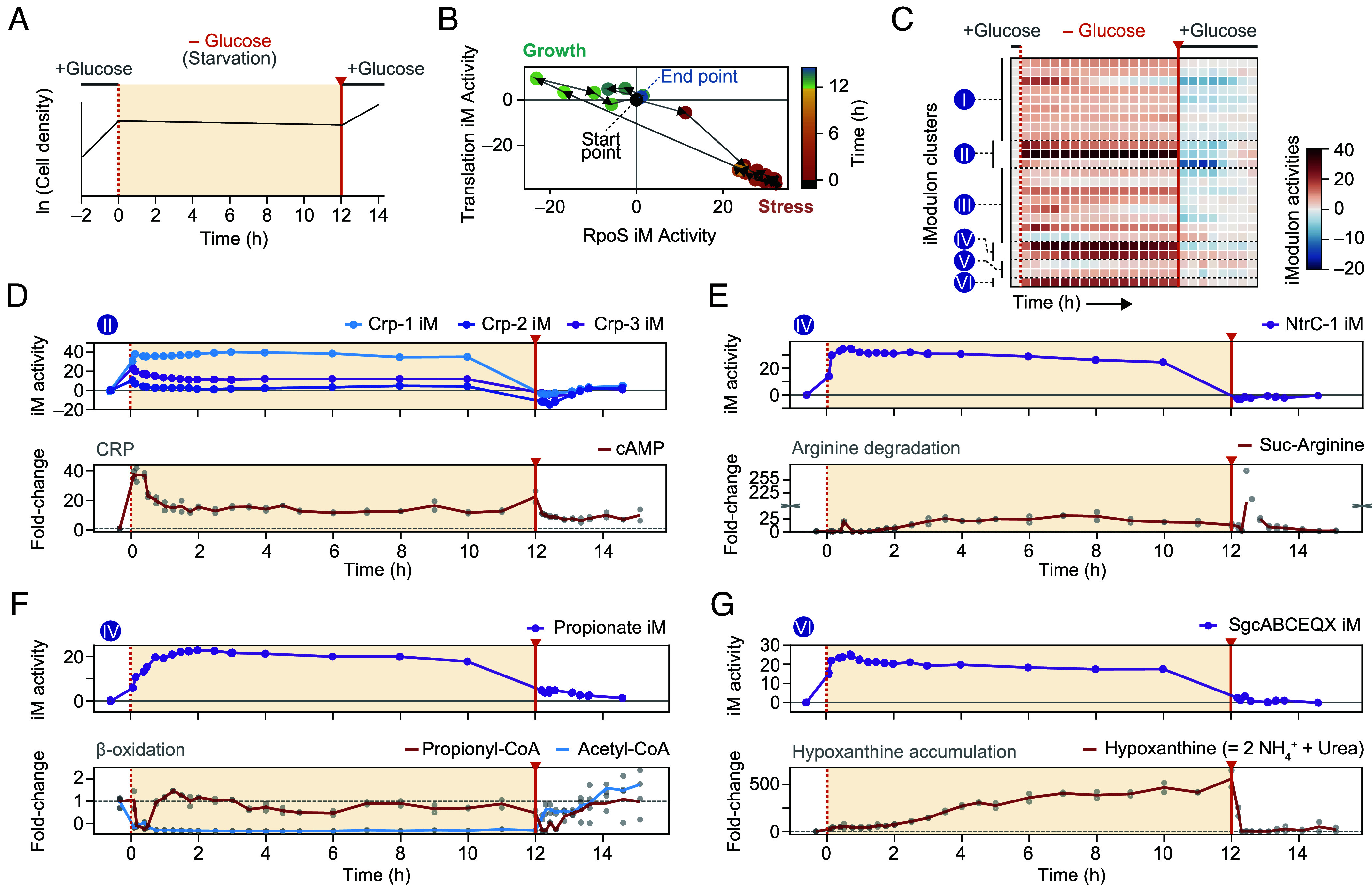
An independent glucose starvation/refeeding time course shows overlapping activation of selected carbon- and stress-associated iModulons. (*A*) Experimental design of published glucose starvation/refeeding experiment (30). *E. coli* cells were grown in M9 medium with glucose, subjected to 12 h of carbon starvation (orange shaded region) via rapid medium exchange, then resupplied with glucose for 2 h of recovery. Cell density is shown on a log scale to indicate growth arrest and resumption. (*B*) Projection of the time-course samples onto the Translation and RpoS iModulon activity space. Translation iModulon vs. RpoS iModulon activities. Points are colored by time (hours). Arrow connects sampled time points in the Translation–RpoS iModulon activity space during starvation and recovery. (*C*) Heat map of selected iModulon activities during the starvation/refeeding time course, grouped according to the iModulon clusters defined in Fig. 1. (*D*) Crp iModulon and cAMP dynamics. *Upper* panel: Activities of Crp-1, Crp-2, and Crp-3 iModulons. *Lower* panel: Intracellular cAMP levels (fold-change relative to prestarvation). Shading marks starvation; orange triangles denote glucose re-addition. (*E*) NtrC-1 iModulon activation and arginine metabolism. *Upper* panel: NtrC-1 iModulon activity. *Lower* panel: N(2)-succinyl-arginine accumulation indicating arginine degradation pathway activation. (*F*) Propionate iModulon and propionyl-CoA dynamics. *Upper* panel: Propionate iModulon activity. *Lower* panel: Propionyl-CoA (red) and acetyl-CoA (blue) levels showing sustained propionyl-CoA despite acetyl-CoA depletion. (*G*) SgcABCEQX iModulon and hypoxanthine accumulation. *Upper* panel: SgcABCEQX iModulon activity. *Lower* panel: Hypoxanthine levels, a purine catabolism product that releases 2 NH_4_^+^ equivalents plus urea upon deamination. Metabolites are shown as fold-change to the pre-starvation sample; dots show time points (replicates in gray), lines show means; beige shading denotes starvation. Data from Lempp et al. (30) were reanalyzed using the PRECISE-NP881 iModulon decomposition.

To examine whether the iModulons identified in the single-carbon atlas are also engaged during an acute nutrient perturbation, we reanalyzed a previously published time-resolved glucose-starvation/refeeding dataset that jointly profiled the *E. coli* transcriptome and metabolome (Fig. 6*A*) (30). This study quantified the in vivo coupling of cAMP to CRP activity and documented rapid macromolecule recycling upon starvation, including transient accumulation of free amino acids and hypoxanthine—signatures of protein and RNA degradation (7, 30). We projected the RNA-seq time series onto the PRECISE-NP881 iModulon structure and integrated matched metabolite trajectories. Projection onto the Translation and RpoS iModulon activity space showed movement from a growth-associated state toward a stress-associated state during starvation, followed by return toward the starting state after glucose refeeding (Fig. 6*B*). Across the iModulon activity heat map, selected modules from clusters II, IV, and VI changed during starvation with overlapping kinetics rather than a strict temporal sequence (Fig. 6*C*).

The module-specific dynamics were consistent with the individual carbon atlas. The three Crp iModulons increased rapidly after glucose removal (Crp-1 *P* < 1.67 × 10^−6^; Crp-2 *P* = 0.03; Crp-3 *P* < 2.04 × 10^−5^ throughout starvation), coincident with a 37.5-fold rise in cAMP (Fig. 6*D*). In parallel, matched metabolomics showed rapid remodeling of glycolysis/gluconeogenesis-associated intermediates during glucose starvation (*SI Appendix*, Fig. S9*A*). Consistent with protein degradation (30), arginine levels initially increased 2.3-fold before being consumed via the AST pathway (*SI Appendix*, Fig. S9*B*). NtrC-1 iModulon activity also increased during starvation (*P* < 2.04 × 10^−5^), coincident with arginine-degradation-associated metabolite changes, including a 31.2-fold increase in N2-succinyl-arginine (Fig. 6*E*). Propionate iModulon activity increased as well (*P* < 2.38 × 10^−5^), while propionyl-CoA remained detectable despite depletion of acetyl-CoA and other central-carbon intermediates (0.61 to 1.35-fold of baseline throughout starvation; Fig. 6*F* and *SI Appendix*, Fig. S9*B*). In contrast, Cluster V modules showed minimal change (|Δactivity| < 7.17; *SI Appendix*, Fig. S9*C*). Finally, hypoxanthine, a purine-catabolism product associated with nitrogen recycling, accumulated 563-fold during starvation, and SgcABCEQX iModulon activity increased (*P* < 1.92 × 10^−6^; Fig. 6*G*). Thus, the starvation/refeeding data show that selected CRP-linked, NtrC-1/Propionate-associated, and SgcABCEQX modules identified in the steady-state atlas are also engaged during acute glucose removal.

Upon glucose refeeding, the activities of these modules moved back toward the prestarvation state, consistent with reversal of the acute starvation response (Fig. 6 *B*–*G*). We therefore interpret this independent dataset as contextual support for the atlas: It shows that several iModulons highlighted by the individual carbon compendium are engaged during a dynamic perturbation and reverse upon carbon restoration. The data support a modular and partially concurrent view of TRN outputs during starvation and recovery.

## Discussion

Understanding how bacteria respond to different carbon sources, and how these responses relate to CCR, has been a long-standing topic in microbial physiology (31, 32). In this study, we compiled a large transcriptome compendium and applied ICA–based systems-level analysis to map observed TRN outputs of *E. coli* MG1655 across 43 individual carbon environments. This analysis yields a quantitative atlas of carbon-responsive transcriptional states, organized into four activity-defined substrate groups. Because the primary dataset is based on steady-state growth in individual carbon-source media, we interpret these groups as a top–down organization of condition-specific TRN outputs, not as a temporal hierarchy of regulatory decisions.

A first outcome is that the classical CCR/CRP axis can be decomposed into statistically separable CRP-linked iModulons that are deployed differently across carbon-source conditions. CRP is a canonical integrator of carbon status and transcription (3, 32), and its binding and regulatory landscape has been extensively characterized (4, 14), including interactions with central-carbon regulators such as Cra (16). In our compendium, three CRP-linked iModulons separate CRP-associated transcription into condition-selective components. This is consistent with a graded and promoter-architecture–dependent CCR system rather than a binary on/off switch (3, 32). This separation also aligns with the idea that cAMP signaling coordinates proteome usage with metabolic demands (7), and with evidence that CRP subregulons can couple to stringent-response states in a context-dependent manner (33).

The atlas also connects carbon-responsive iModulons to global growth/stress physiology and model-estimated proteome allocation. Proteome allocation theory and growth laws predict that poor substrates can require larger expression and maintenance costs, leaving fewer resources for translation and rapid growth (6, 9, 34, 35). Consistent with this, Translation- and stress-linked iModulons, including RpoS, showed strong anticorrelation across the individual carbon conditions, capturing the inverse relationship between growth-associated Translation activity and stress-associated RpoS activity. In addition, the observed coupling between carbon limitation, stringent signaling, and growth control is compatible with the central role of (p)ppGpp in global physiology (5, 36, 37) and with reported links between CRP activity and stringent-response networks (33).

A second outcome is that the NtrC-1 and Propionate iModulons mark a subset of carbon-source conditions consistent with propionyl-CoA-associated metabolic stress. The methylcitrate cycle is a recognized route for propionyl-CoA conversion and can be important for bacterial physiology in multiple contexts (19). In our data, iModulons related to arginine/GABA/fatty-acid catabolism and methylcitrate-cycle genes were significantly activated in selected TCA-entry and amino acid-associated conditions, and Δ*prpC* showed growth defects specifically under those conditions. These observations support the interpretation that propionyl-CoA-associated metabolism becomes conditionally relevant. This interpretation is also consistent with known catabolite-repression coupling to propionate catabolic genes (21) and with the biochemical principle that propionyl-CoA can inhibit key dehydrogenase activities (20).

A third outcome is that the prophage-embedded SgcABCEQX iModulon marks a narrow subset of nitrogen-containing slower-growth conditions. Prior work indicates that cryptic/defective prophages can contribute to stress-adaptive functions and can rewire host regulation (38, 39). In this context, we interpret *sgcABCEQX* induction primarily as a marker of a condition-specific transcriptional state that emerges under a subset of nitrogen-containing slower-growth substrates. The absence of growth defects in Δ*sgc* mutants under the assayed conditions argues against a required role in the tested individual carbon growth phenotypes. In an independent starvation/refeeding dataset, *sgcABCEQX* induction coincides with hypoxanthine accumulation (30), consistent with nitrogen recycling linked to purine catabolism. Annotations and coactivation patterns suggest possible connections to unusual sugar/polyol chemistry (26, 40), but direct biochemical validation is required.

The conservation analysis further shows that representative module-associated gene sets differ in their distribution across *E. coli* phylogroups. CRP-linked and Propionate iModulon gene sets were more broadly conserved, whereas dmlA + SCFA iModulons and *sgcABCEQX*-associated gene sets showed more lineage-specific retention (*SI Appendix*, Fig. S7*D*). This is compatible with ecological differentiation across animal-associated and environmental reservoirs (41, 42) and with domestication/retention dynamics of defective prophages (39). Thus, the atlas can help distinguish broadly conserved carbon-response components from more niche-associated modules.

Finally, reanalysis of an independent starvation/refeeding dataset showed that several carbon- and stress-associated iModulons identified in the steady-state atlas are also engaged during an acute perturbation. However, the observed responses were overlapping rather than strictly sequential. We therefore interpret the time course as contextual support for the atlas, not as evidence for a universal temporal hierarchy of carbon-response modules. A direct test of substrate prioritization or coutilization would require mixed-substrate experiments with time-resolved substrate uptake, flux measurements, and dynamic transcriptomics, ideally under conditions where flux and growth rate can be controlled independently.

In summary, this study provides a quantitative systems-level atlas of carbon-response transcriptional states in *E. coli* across diverse individual carbon-source environments. By organizing 25 carbon-catabolism iModulons into four activity-defined substrate groups and integrating growth phenotyping, metabolic modeling, targeted mutants, model-estimated proteome allocation, phylogroup conservation, and an independent starvation/refeeding dataset, we systematize carbon-responsive physiology at scale. This atlas provides a compact reference for interpreting nutrient-responsive transcriptomes and for generating testable hypotheses about how carbon chemistry, growth state, and stress physiology shape *E. coli* behavior across nutritional environments.

## Materials and Methods

### Bacterial Strains and Growth Conditions.

The WT reference strain for all experiments was *E. coli* K-12 MG1655. Routine propagation was carried out at 37 °C either in LB medium or in M9 minimal medium supplemented with a sole carbon source. M9 contained 47.75 mM Na_2_HPO_4_, 22.04 mM KH_2_PO_4_, 8.56 mM NaCl, 18.70 mM NH_4_Cl, 2 mM MgSO_4_, 0.1 mM CaCl_2_, and 0.5 mL L^−1^ of a 2,000× Sauer trace-element solution [100 mM FeCl_3_, 9.54 mM ZnCl_2_, 8.41 mM CoCl_2_, 8.27 mM Na_2_MoO_4_, 0.75 mM CaCl_2_, 0.91 mM CuCl_2_, and 0.5 mM H_3_BO_3_ in 3.7% (w/w) HCl]. Unless stated otherwise, chemicals were obtained from Sigma-Aldrich.

Forty-three carbon substrates spanning sugars, acids, polyols, amino acids, and nucleosides were chosen according to two criteria: i) *E. coli* displayed robust growth on the compound [Z-score > 6.5 relative to the negative control in PM-kBase (https://omnilogdb.org/)] and ii) commercial availability (Dataset S1). Each carbon substrate was supplied at 0.5% w/v; the full list includes D-glucose, D-glucose-6-phosphate, D-galactose, L-arabinose, D-gluconate, maltotriose, maltose, N-acetyl-neuraminic acid, N-acetyl-D-glucosamine, L-fucose, D-glucuronate, D-galacturonate, L-lyxose, D-lactose, D-saccharate, methylpyruvate, D-mannitol, uridine, dulcitol, pyruvate, D-sorbitol, L-rhamnose, D-serine, mucate, D-melibiose, β-methyl-D-galactoside, D-trehalose, D-mannose, cytidine, L-serine, D-alanine, D-glucosamine, L-aspartate, α-ketoglutarate, m-tartarate, DL-malate, L-proline, L-asparagine, inosine, and adenosine. Cultures were shaken aerobically until mid-exponential phase (OD_600_ ≈ 0.25 to 0.50), at which point samples were harvested. Triplicate cultures were used for growth-rate measurements unless noted otherwise.

### RNA Extraction and RNA-Seq Library Construction.

Six milliliters of culture were mixed with 12 mL RNAprotect Bacteria Reagent (Qiagen), vortexed for 5 s, incubated for 5 min at ambient temperature, and centrifuged (12,000×*g*, 10 min). Pellets were flash-frozen (−80 °C) before extraction. Total RNA was purified with the Quick-RNA Fungal/Bacterial Microprep kit (Zymo) as described previously (43, 44). Concentration and integrity were determined with a NanoDrop spectrophotometer and an Agilent 4150 TapeStation. One microgram of RNA was depleted of rRNA and genomic DNA using the RiboRid protocol with *E. coli*-specific probes (45); depletion success was verified on the TapeStation. Directional libraries were prepared with the NEBNext Ultra II kit (New England Biolabs), quality-checked on a TapeStation (D1000 ScreenTape), and quantified with a Qubit dsDNA HS assay. Equimolar pools were sequenced on an Element AVITI instrument (Element Biosciences; 2 × 150 bp) at The Scripps Research Institute Genomics Core.

### RNA-Seq Data Processing.

Read processing followed the iModulonMiner workflow (46). Briefly, adaptor-trimmed reads were aligned to the MG1655 genome (NC_000913.3), counted per gene, and converted to TPM. Biological replicates were highly concordant (R^2^ > 0.95); log_2_(TPM + 1) values formed the input matrix for downstream analysis. After quality control, the PRECISE-NP881 compendium comprised 881 transcriptomes—346 from this study and 535 from PRECISE-1 K (12).

### ICA.

RNA-seq profiles were normalized by log_2_-TPM of two reference cultures grown on 0.2% glucose in exponential phase, thereby defining the glucose condition as the baseline. Robust independent signals were then extracted with the PyModulon implementation of FastICA (46). FastICA was executed 100 times with random initializations, and the resulting components were clustered with DBSCAN to retain only those reproduced across runs.

For each consensus component, genes whose weights significantly deviated from a normal distribution (D’Agostino K^2^ test) were assigned to the corresponding iModulon; component signs were flipped, where necessary, so that the majority of member genes carried positive weights.

Gene-set enrichment against RegulonDB was performed with Fisher’s exact test and Benjamini–Hochberg correction (FDR ≤ 1 × 10^−5^). Following published criteria (12, 47), iModulons were labeled as Regulatory, Biological, Technical, or Uncharacterized. In this manuscript, “iModulon” refers to a statistically independent gene-expression component with significant gene weights and condition-specific activities inferred by ICA. Differential iModulon activity between conditions was assessed by comparing the absolute difference in mean activity to the component’s log-normal activity distribution; modules with |Δactivity| > 5 and FDR below the chosen cutoff were regarded as significantly altered.

### Growth Rate Measurements.

Starter cultures grown on glucose minimal medium were washed and inoculated into M9 containing each test carbon. Flask OD_600_ was measured on a BioMate 3S spectrophotometer (Thermo Fisher Scientific); microplate assays used an Infinite 200 Pro reader (100 µL per well, 37 °C, orbital shaking, 10 to 15 min intervals; Tecan). Specific growth rates (µ) were obtained from the log-linear phase by linear regression using QurvE (48). At least three biological replicates were run per condition, and the mean μ was used for comparisons.

### Knock-Out Mutants.

Targeted gene knockout strains were used to test whether selected module-associated genes contributed to growth under representative substrate conditions. We obtained *ΔprpC*, *ΔastC*, *ΔsgcC*, and *ΔsgcX* single-gene deletion mutants from the KEIO collection (49). The BW25113 parental strain was used as the control for these mutants. All mutant and control strains were cultured in M9 minimal medium under selected carbon-source conditions chosen to test the physiological relevance of the corresponding module-associated genes.

### Genome-Scale Metabolic Modeling.

Flux balance analysis was carried out in Python with COBRApy (50) using the *E. coli* genome-scale model iML1515 (51) obtained from the BiGG database in a standard manner (52). To enable cross-substrate comparisons for carbon sources that differ in size and carbon number, we fixed total carbon influx (C_in) across substrates by scaling each substrate’s molar uptake bound by its carbon number (v_uptake = −C_in/nC). Unless otherwise stated, simulations used C_in = 60 C-mmol gDW^−1^ h^−1^ and an oxygen uptake lower bound of −20 mmol gDW^−1^ h^−1^ to define a standardized reference condition. Carbon fate was summarized by partitioning imported carbon into i) inferred biomass conversion (C_in − total carbon export) and ii) carbon dissipation via CO_2_ and exported metabolites.

### Comparative Conservation Across Phylogroups.

Representative *E. coli* genomes were grouped by Clermont phylogroups A, B1, B2, C, D, E, and F (n = 10,511) by in-silico Clermont typing (EzClermont/ClermonTyping) (23); assemblies with EC_control_fail or Cryptic labels were excluded. For each gene set (CRP, Propionate iModulon, dmlA iModulon, SCFA iModulon, SgcABCEQX iModulon), proteome-wide searches were run with DIAMOND (blastp, outfmt 6) and the best hit per gene was retained per strain. Unless stated otherwise, search filters were e-value ≤ 1 × 10^−5^, percent identity ≥ 70%, and alignment coverage ≥ 80% of the query length (24, 53). Per-strain AAI (%) was computed as a length-weighted identity across genes in the set, keeping one best hit per gene and assigning zero to missing genes: AAI (%) = 100 × Σ_i_ [(protein identity_i_/100) × alignment length_i_]/Σ_i_ query length_i_. This adapts the widely used AAI concept (average AAI of shared orthologs) to an iModulon-level score (27). We summarized conservation as “conserved” when AAI ≥ 95% with Wilson 95% CIs per phylogroup. To contextualize conservation vs. overall relatedness, we computed Mash distances from each genome to MG1655 (25); for closely related genomes Mash distance *D* tracks ANI well and can be interpreted approximately as ANI (%) ≈ (1 − D) × 100.

### Omics-Constrained ME Model Simulations.

Proteome allocation followed the previous study (54) using the FoldME model (55) implemented in COBRAme (56). Experimental growth rates constrained the model; expression data were integrated as described previously (54), and steady-state solutions provided protein mass fractions assigned to carbon utilization-related and RpoS iModulons. Proteome sectors that were constrained using expression data include: Crp-1/2/3, NtrC-1, Propionate, DmlA, SCFA, SgcABCEQX, ppGpp, Pentose Phosphate Pathway, TCA Cycle, Oxidative Phosphorylation, and Glycolysis.

### Reanalysis of Starvation/Refeeding Time-Course Data.

We integrated a published time-course that jointly profiled the *E. coli* transcriptome and metabolome during a growth → 12 h carbon starvation → 2 h recovery sequence in a bioreactor. Raw and processed data were obtained from GEO (GSE131992; RNA-seq) and MetaboLights (MTBLS1044; LC-MS/MS metabolomics), together with the authors’ Source Data files (30). RNA-seq reads were processed using our standard pipeline, and gene expression values were projected onto the PRECISE-NP881 iModulon decomposition matrix using PyModulon (46). Specifically, activities A(t) were inferred with the “infer_activities” routine using our fixed gene-weight matrix M (signs oriented as in the compendium), after aligning gene identifiers. Metabolomics data were extracted from supplementary files. Key metabolites, including glycolytic intermediates (DHAP, FBP, hexose-P, PEP), TCA cycle components (citrate, malate, fumarate, succinate, succinyl-CoA, acetyl-CoA), propionyl-CoA, amino acids (arginine, N2-succinyl-arginine), purines (hypoxanthine), and cAMP were tracked across all time points.

### Statistical Analysis.

Unless noted, experiments employed three biological replicates and results are given as mean ± SD. Statistical tests were two-sided and carried out in Python (SciPy, StatsModels) or GraphPad Prism v10.

## Supplementary Material

Appendix 01 (PDF)

Dataset S01 (XLSX)

Dataset S02 (XLSX)

Dataset S03 (XLSX)

## Data Availability

RNA-seq; PRECISE-1K matrices, metadata, and raw reads; PRECISE-NP881 matrices and metadata; and Code (FBA and ME simulations; ME-model constraints/solving; ICA/iModulon analysis; and data processing) Scripts for ICA, iModulon analysis, and data processing and New RNA-seq data for carbon perturbations data have been deposited in NCBI SRA [PRJNA1273304 (57) and PRJNA1273601 (58)], GitHub (SBRG/precise1k) and NCBI GEO [https://github.com/SBRG/precise1k (59)]. GEO accession numbers are listed in the metadata file located in the GitHub repository at the path: data/precise1k/metadata_qc.csv), Dataset S2, and GitHub [https://github.com/SBRG/cobrame (60), https://github.com/SBRG/ecolime (61), https://github.com/SBRG/solvemepy (56, 62, 63), https://github.com/SBRG/ME-script (64), https://github.com/SBRG/iModulonMiner (65), and https://github.com/SBRG/pymodulon (46, 66)], respectively. All other data are included in the manuscript and/or supporting information.
