# Supplementary material for: A systems-level atlas of carbon-response transcriptional states in Escherichia coli
Source: Proc Natl Acad Sci U S A. 2026 Jul 1;123(27):e2531884123. doi: 10.1073/pnas.2531884123 (PMC13343008; doi:10.1073/pnas.2531884123)
Supplement: Supplementary file 1 — Appendix 01 (PDF) [file pnas.2531884123.sapp.pdf]

PNAS

Supporting Information for
A systems-level atlas of carbon-response transcriptional states in *Escherichia coli*

Jongoh Shin[1,2,3,*], Arjun Patel[1], Xuwen A. Lou[1], Edward Alexander Catoiu[1], Jayanth Krishnan[1], Ying Hefner[1], Richard Szubin[1], Jaemin Sung[1], Hyeoncheol Francis Son[4], Daniel C. Zielinski[1], and Bernhard Ørn Palsson[1,5,6,*]

[1]Department of Bioengineering, University of California San Diego, La Jolla, CA, 92093, USA.
[2]Department of Biological Sciences, Chonnam National University, Gwangju, 61186, Republic of Korea.
[3]Institute of Synthetic Biology for Carbon Neutralization, Chonnam National University, Gwangju, 61186, Republic of Korea
[4]School of Biological Sciences and Technology, Chonnam National University, Gwangju, 61186, Republic of Korea.
[5]Novo Nordisk Foundation Center for Biosustainability, Technical University of Denmark, Lyngby, 2800, Denmark.
[6]Department of Pediatrics, University of California, San Diego, La Jolla, CA, USA.

* To whom correspondence should be addressed.

Email: joshin@jnu.ac.kr and bpalsson@ucsd.edu

This PDF file includes:

Figures S1 to S9
Legends for Dataset S1 to S3

Other supporting materials for this manuscript include the following:

Dataset S1 to S3

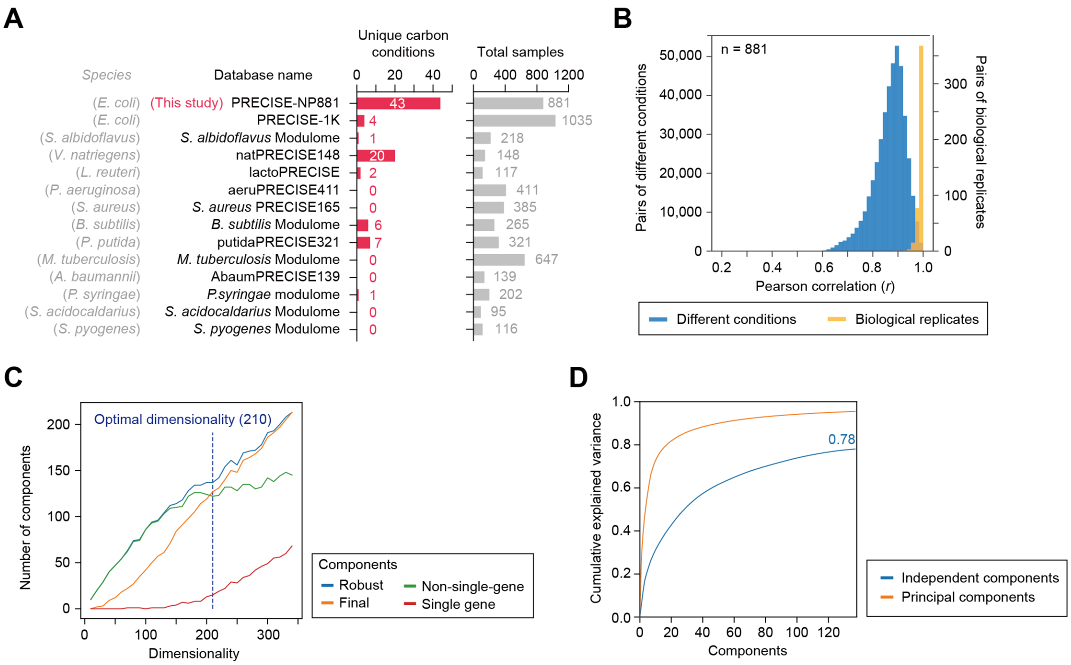

Fig. S1. Dataset comparison, quality control, and ICA dimensionality selection. (A) Benchmark of the PRECISE-NP 881 compendium against 12 published bacterial transcriptome resources. Red bars indicate the number of unique carbon-source conditions; grey bars indicate total samples. **(B)** Reproducibility of the PRECISE-NP 881. Histogram of pairwise Pearson correlations for expression profiles derived from different conditions (blue) and biological replicates of the same condition (yellow). **(C)** Selection of the optimal dimensionality for independent component analysis (ICA). The number of robust (blue), non-single-gene (green), single-gene (red), and final (orange) components is plotted against tested dimensionalities; 210 components (dashed line) were selected. **(D)** Cumulative variance explained by the final 137 independent components (blue) compared with principal components (orange); ICA captures 78 % of the total expression variance with far fewer components.

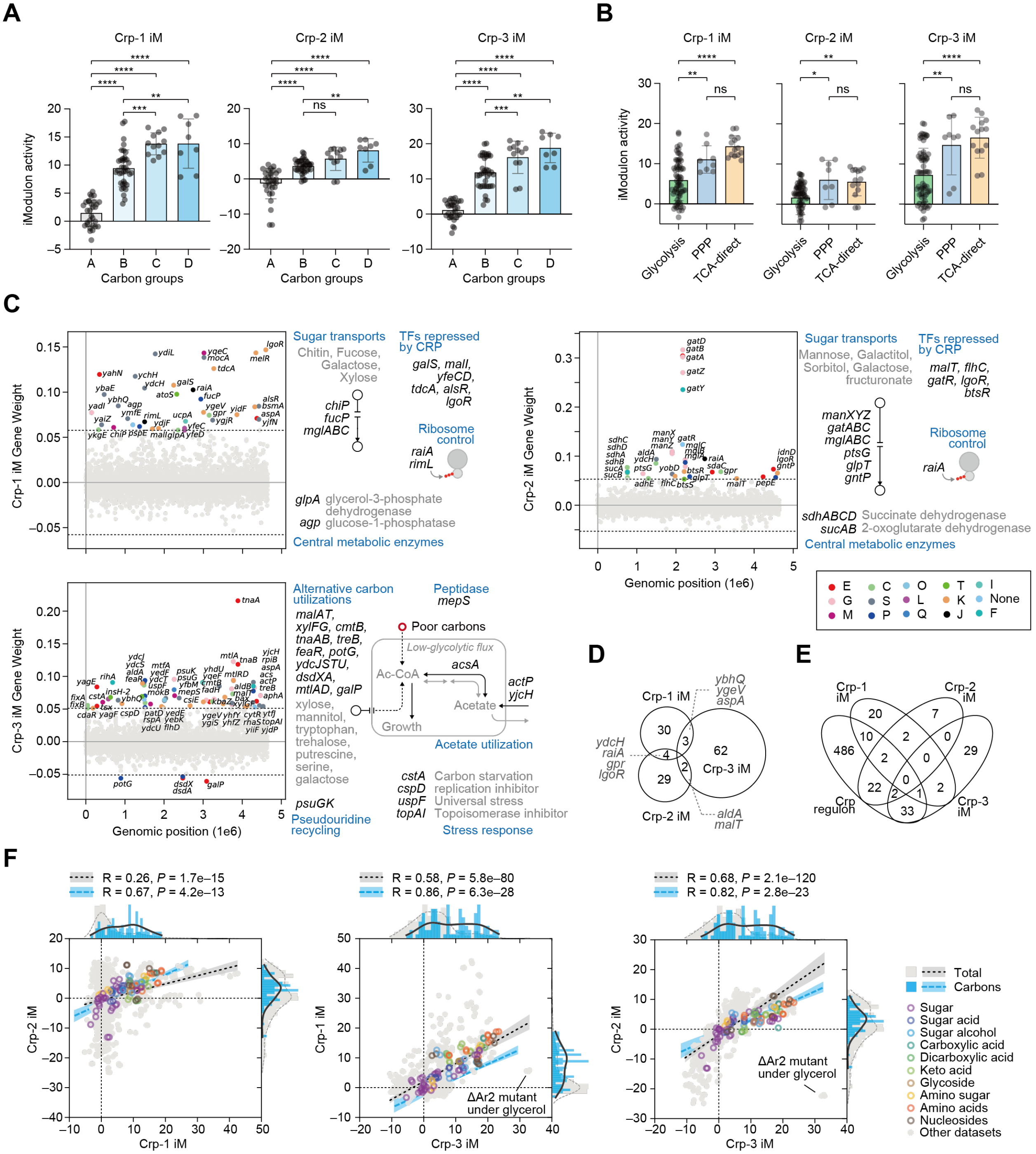

Fig. S2. Distinct gene membership of CRP iModulons and their activities across various carbon conditions. (A) Activity of Crp iMs on Group A–D (please refer to **Figure 1**). Bars indicate means ± SD, with individual replicates shown as points. Multiple comparisons revealed significant differences in the activity of Crp iMs among the carbon groups (one-way ANOVA with post hoc tests; $**P < 0.01$; $***P < 0.001$; $****P < 0.0001$). **(B)** Crp-iM activities for carbon substrates entering glycolysis, the pentose phosphate pathway (PPP), or the TCA cycle directly. Bars indicate means ± SD, with individual replicates shown as points. Multiple comparisons revealed significant differences in the activity of Crp iMs (one-way ANOVA with post hoc tests; $*P < 0.05$; $**P < 0.01$; $****P < 0.0001$). **(C)** Scatter plots of gene weights along the *E. coli* genome for Crp-1, Crp-2, and Crp-3 iModulons. Genes above threshold are colored by functional category (legend, right) and annotated with representative names. Insets summarize gene functions as Clusters of

Orthologous Genes (COGs) for each iModulon. **(D–E),** Venn diagram illustrating the overlap among gene memberships in the **(D)** three Crp iModulons and **(E)** previously known Crp regulon. Numbers indicate how many genes are uniquely or jointly assigned, with representative genes shown in each region. **(F)** Scatter plots showing the correlations between the activities of the Crp-iModulon pairs. Each point corresponds to a single condition, colored by the carbon substrates category. Pearson correlations are provided for the total dataset (gray) and for the carbon subset (colored). The "ΔAr2 mutant under glycerol" is also highlighted, indicating a notable outlier.

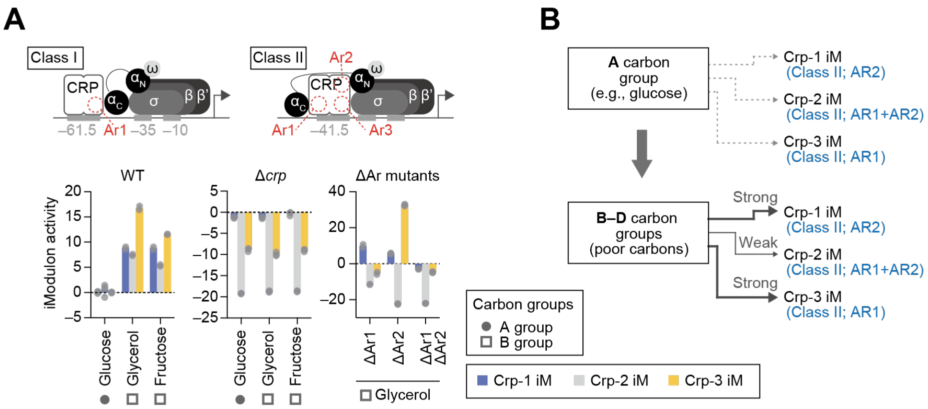

Fig. S3. CRP promoter-architecture context and Crp iModulon activities under representative carbon conditions. (A) Schematic representation of Class I and Class II Crp-dependent promoters, illustrating CRP's three activation regions (AR1, AR2, AR3) with core RNAP binding sites. Bar plots show the activities of Crp-1, Crp-2, and Crp-3 iModulons for the wild-type (WT), ΔCrp, and ΔAr (AR1, AR2, AR1+AR2) mutant strains grown in glucose, glycerol, or fructose. Loss of Crp or individual activation regions leads to differential effects on each Crp iModulon, consistent with differential sensitivity of these CRP-linked iModulons to CRP activation-region perturbations. **(B)** Conceptual summary of Crp-1, Crp-2, and Crp-3 iModulons usage under standard glucose and Groups B-D carbon-source conditions. Crp-1 and Crp-3 iModulons show stronger activity shifts than Crp-2 across these conditions.

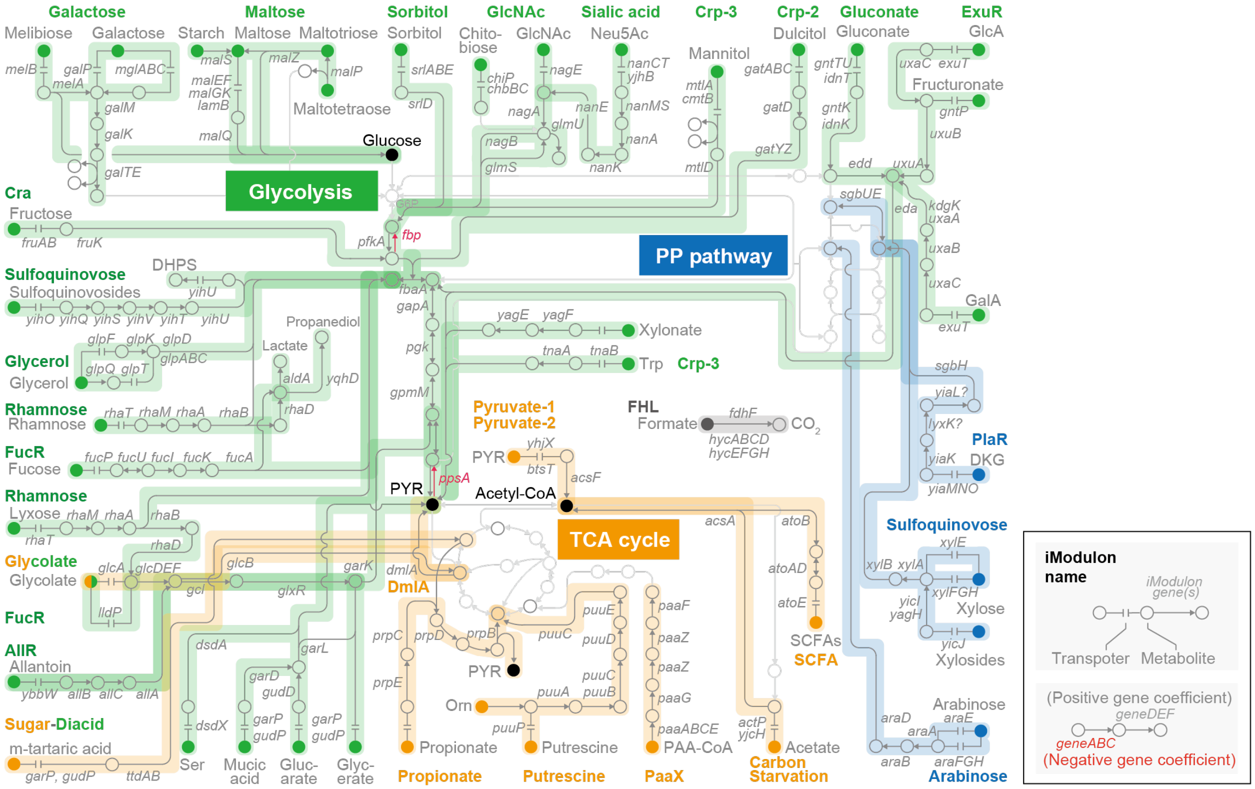

Fig. S4. Integrated map of *E. coli* carbon catabolism iModulon. The map illustrates how diverse uptake routes funnel into a limited set of central pathways that are coordinated by distinct, yet partially overlapping, iModulons. Pathways used by the 43 carbon substrates are overlaid on a simplified metabolic network. Lines represent enzymatic steps; circles, intracellular metabolites. Gene sets belonging to the same catabolic iModulon (labelled at entry points) are colored by their principal metabolic destination: glycolysis (green), pentose-phosphate pathway (blue), or the TCA cycle/short-chain-fatty-acid branch (orange). Bold black metabolite symbols mark key convergence nodes (PYR, pyruvate; Acetyl-CoA). Gene names are shown in grey when their ICA coefficient is positive and in red when negative, highlighting opposing regulation within the same module.

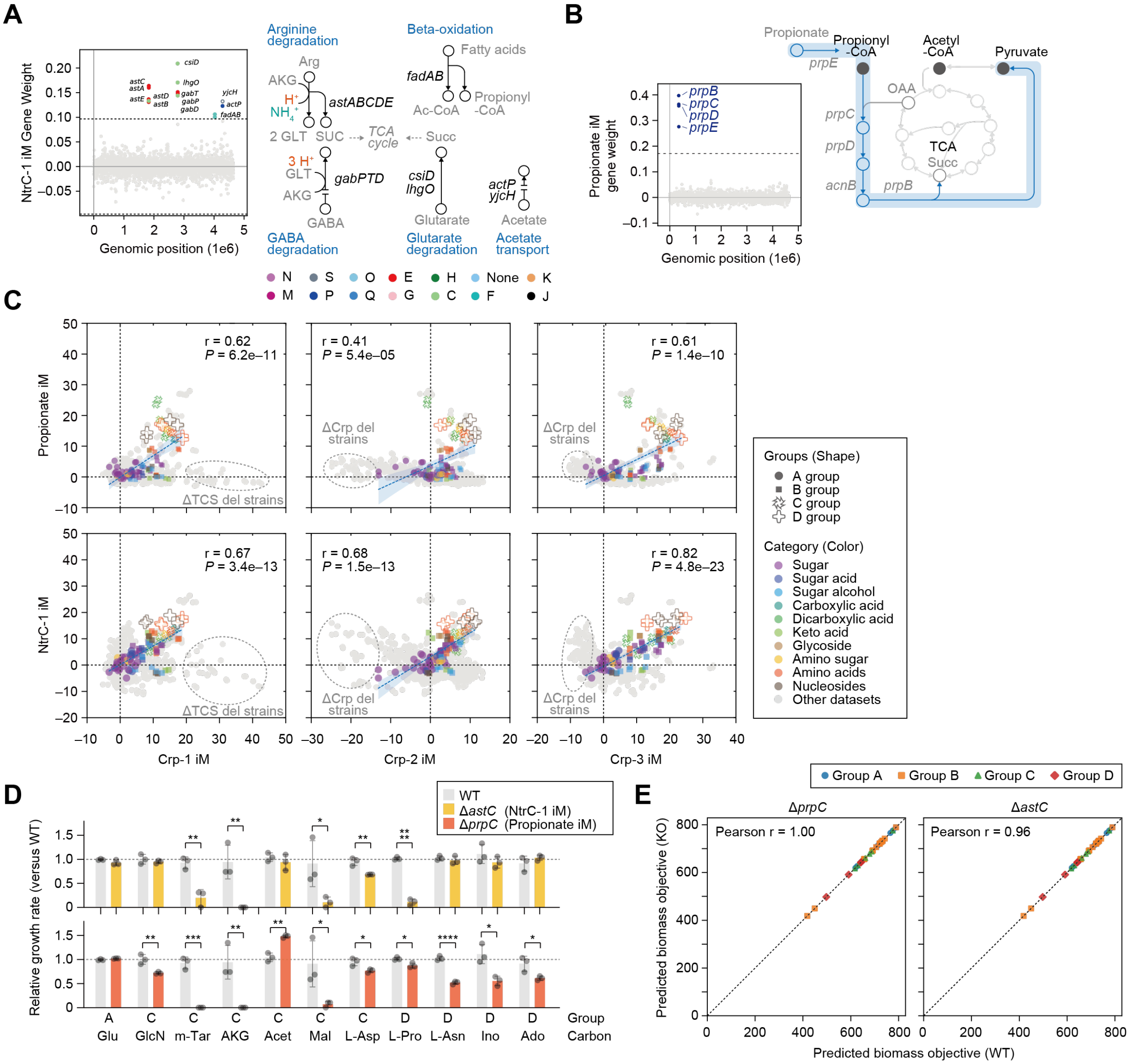

Fig. S5. Propionate and NtrC-1 iModulon gene membership and their activity relationship with Crp iModulons. (A) Genomic distribution of the NtrC-1 iModulon genes. Left, gene weights (y-axis) plotted against chromosomal position (x-axis) for all *E. coli* genes; the horizontal dashed line marks the iModulon inclusion threshold. Genes exceeding the threshold are color-coded by COG functional category (legend) and labeled; key metabolic blocks are annotated in the schematic. Right, the schematic to the right summarizes the constituent routes: arginine degradation (Arg → α-ketoglutarate, AKG), γ-aminobutyric-acid degradation (GABA → succinate, Succ), glutarate degradation (Glu → Succ), β-oxidation of fatty acids (acetyl-CoA, Ac-CoA, and propionyl-CoA intermediates), and acetate transport. Dashed arrows converge on the tricarboxylic-acid (TCA) cycle. **(B)** Genomic distribution of the Propionate iModulon genes. As in **(A)** panel, but for the Propionate iModulon. The four-gene *prpBCDE* operon, which channels propionate to pyruvate via propionyl-CoA, dominates the module and is highlighted. OAA, oxaloacetate; Pyr, pyruvate. **(C)** Correlation between Propionate or NtrC-1 iModulon activity and the three Crp iModulons. Points are colored by carbon-source class (legend) and shaped by carbon group (A–D). Pearson correlation coefficients (r) and corresponding two-tailed *P*-values are shown. **(D)** Growth

phenotypes of representative deletion mutants. Relative growth rates of Δ*astC* (representative of the NtrC-1 iModulon) and Δ*prpC* (representative of the Propionate iModulon) mutants, compared with *E. coli* BW25113 wild type (grey), in M9 medium supplemented with the indicated carbon substrates. Bars represent the mean of three biological replicates ± s.d. Asterisks indicate significant differences from wild type (Welch's t-test: $P < 0.05$ *, $P < 0.01$ **, $P < 0.001$ ***, $P < 0.0001$ ****). *ΔastC* and *ΔprpC* mutants are from the KEIO collection. Glu, Glucose; GlcN, N-Acetyl-D-glucosamine; m-Tar, m-Tartaric acid; AKG, α-Ketoglutaric acid; Acet, Acetate; Mal, Malate; L-Asp, L-Aspartic acid; L-Pro, L-Proline; L-Asn, L-Asparagine; Ino, Inosine; Ado, Adenosine. **(E)** FBA knockout. Predicted maximal growth for WT versus Δ*prpC* (left) and WT versus Δ*astC* (right) under standardized carbon-normalized uptake bounds (C_in = 60 C-mmol gDW^{-1} h^{-1}; O_2 LB = −20 mmol gDW^{-1} h^{-1}). Steady-state FBA predicts minimal/no growth reduction upon deletion, consistent with the interpretation that the experimental defects reflect propionyl-CoA stress mechanisms not represented in stoichiometric FBA.

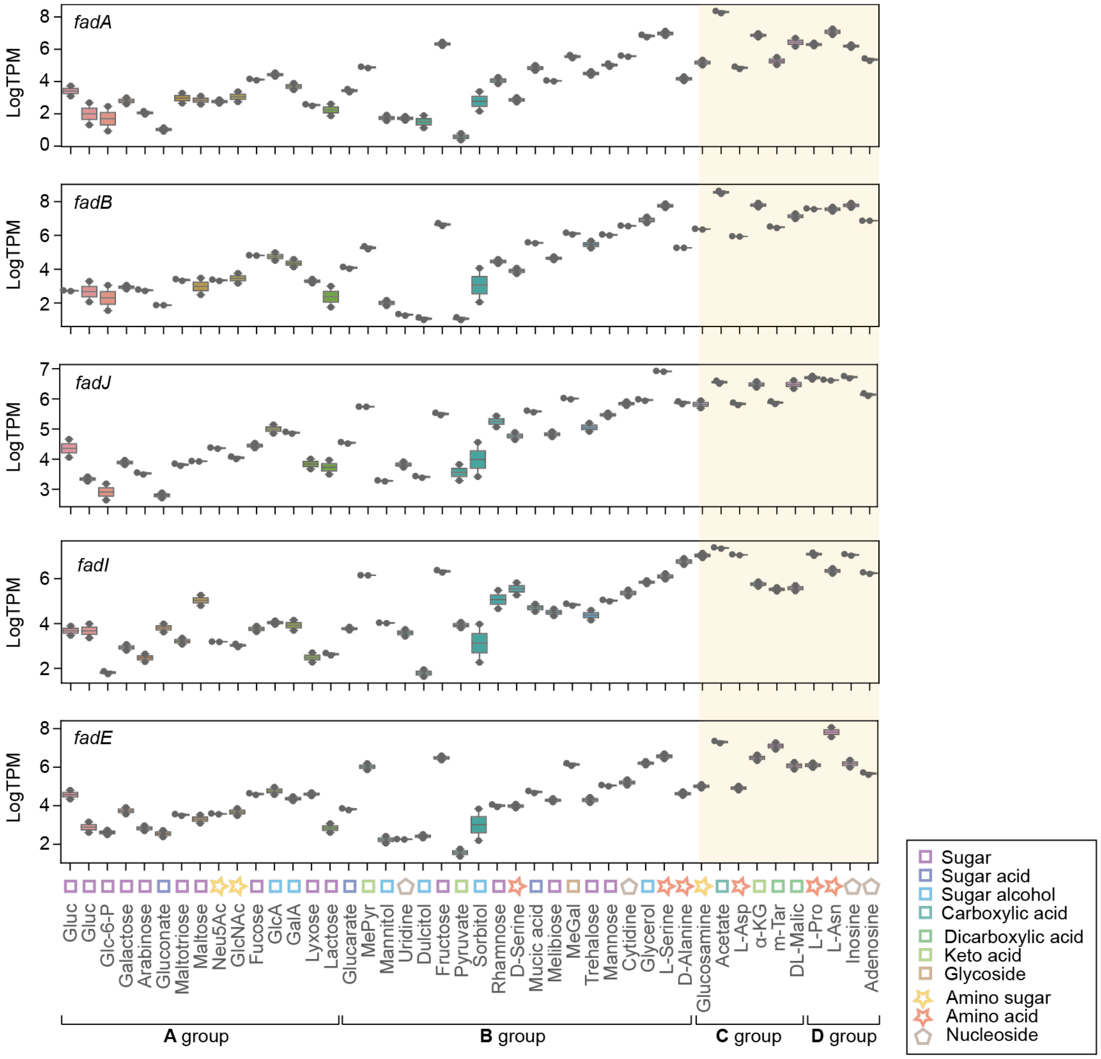

Fig. S6. Expression levels of fatty acid catabolism genes (*fadA*, *fadB*, *fadJ*, *fadI*, *fadE*) under diverse carbon substrates. Shown are logTPM values for each gene across the four activity-defined substrate groups (**A**–**D**) defined in **Figure 2**, with individual carbon substrates. The shaded region highlights groups C and D, where these fatty acid degradation genes tend to exhibit higher expression, consistent with increased expression of fatty-acid catabolism genes in Groups C/D, which include many slower-growth, TCA-entry and amino-acid-associated conditions. Gluc, D-Glucose; Glc-6-P, D-Glucose-6-phosphate; Neu5Ac, N-Acetyl-neuraminic acid; GlcNAc, N-Acetyl-D-glucosamine; GlcA, D-Glucuronic acid; GalA, D-Galacturonic acid; MePyr, Methylpyruvate; MeGal, β-Methyl-D-galactoside; L-Asp, L-Aspartic acid; α-KG, α-Ketoglutaric acid; m-Tar, m-Tartaric acid; DL-Malic, D-Malic acid; L-Pro, L-Proline; L-Asn, L-Asparagine.

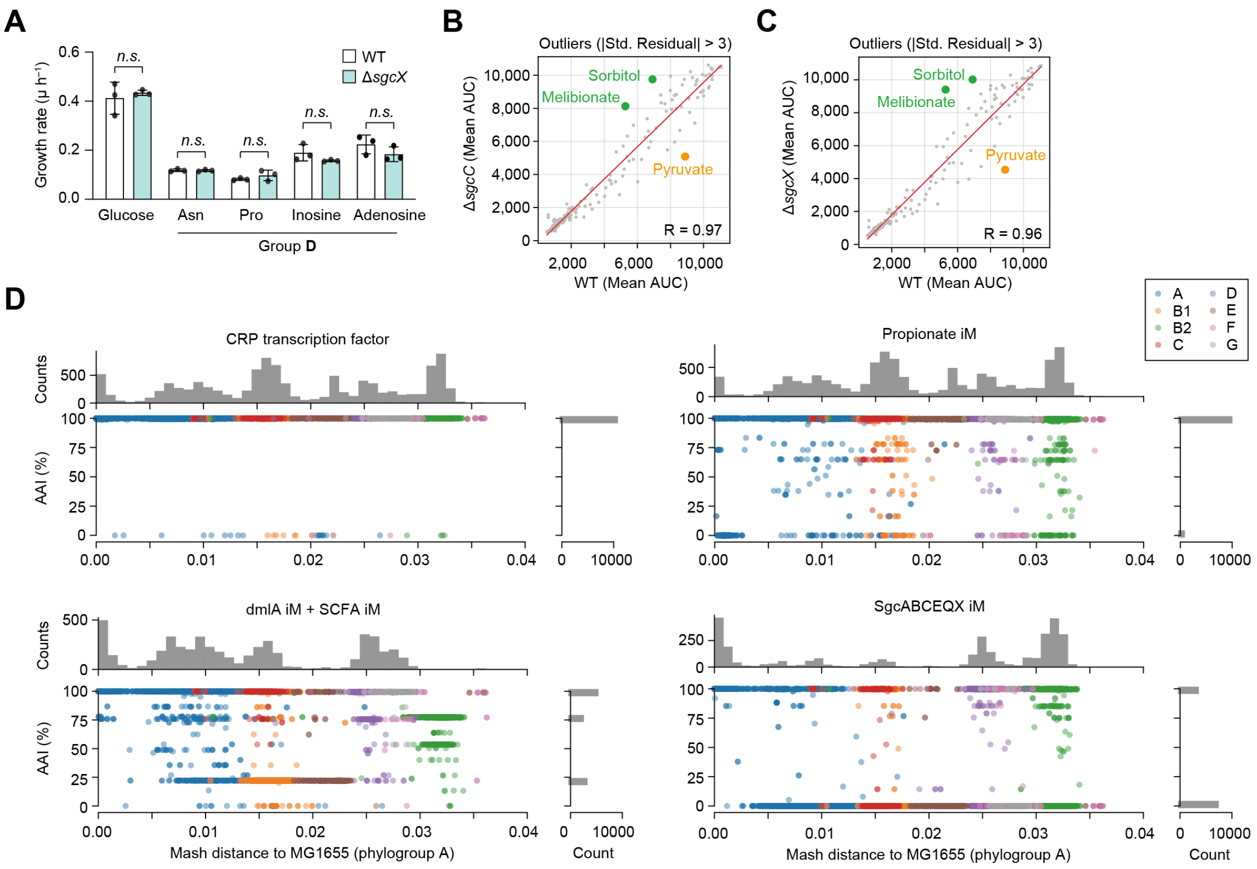

Fig. S7. High-throughput carbon-utilization profiling of *sgc* operon mutants. (A) Relative growth rate of Δ*sgcX* mutants compared with wild-type (WT) on Group D substrates (mean ± s.d., n = 3). Welch's t-test detects no significant difference (n.s.), indicating that the operon is dispensable for the utilization of Group D substrates. Pro, L-Proline; Asn, L-Asparagine. **(B–C),** Scatter plots comparing the growth (mean Area Under the Curve, AUC) of (b) Δ*sgcC* and (c) Δ*sgcX* mutants from the KEIO collection against the *E. coli* BW25113 wild-type (WT) strain across 190 different carbon substrates from Biolog PM1 and PM2A plates. Each point represents a single carbon substrate. The red line indicates the linear regression, showing a high correlation between mutant and WT growth overall (R = 0.97 for Δ*sgcC*; R = 0.96 for Δ*sgcX*). Outliers (Standardized Residual > 3) are highlighted. Outside these outliers, both mutants behave like WT, indicating that the *sgcABCDEQX* operon is dispensable for the utilization of most PM1/PM2A carbon substrates. **(D)** Amino-acid identity (AAI) of iModulon genes versus genomic divergence across high-quality *Escherichia coli* assemblies. Each dot is one assembly, colored by Clermont phylogroup (legend). The x-axis shows the Mash distance to MG1655. The y-axis is Operon-AAI (%), computed as a length-weighted identity across genes in the set: $AAI\ (\%) = 100\ \times \frac{\sum_i\ (protein\ identity_i/100)\times alignment\ length_i}{\sum_i\ query\ length_i}$, keeping the best hit per gene and treating missing genes as zero contribution. Top marginal histograms summarize the distance distribution among strains with AAI ≥ 95% in each panel; right-side histograms show counts with the horizontal axis fixed at 0–10,000 counts for comparability. Panels show CRP protein, propionate iModulon, dmlA iModulon, SCFA iModulon, and SgcABCEQX iModulon genes queried against a comprehensive *E. coli* proteome database using DIAMOND protein alignments.

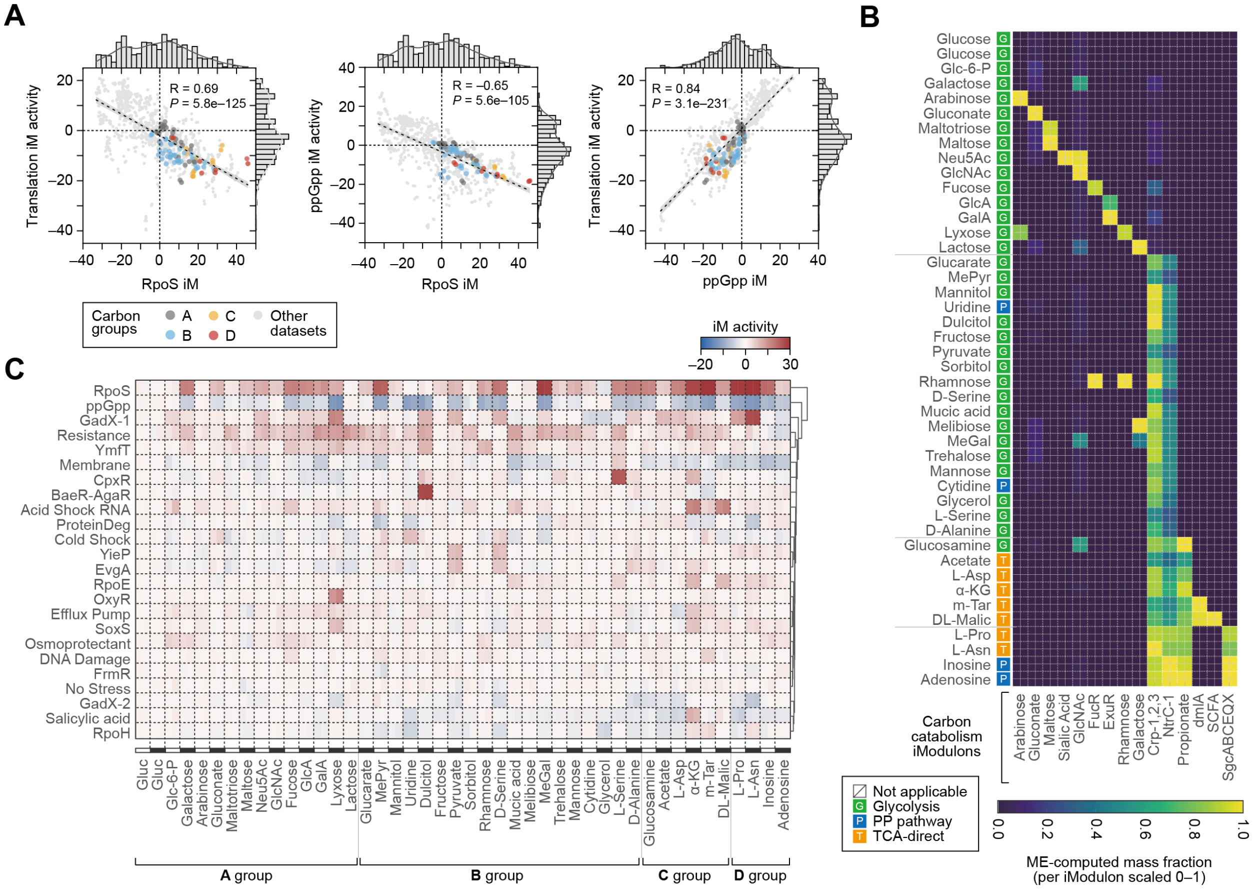

Fig. S8. Relationships among growth-associated, stress-associated, and model-estimated carbon-catabolism sectors. (A) Pairwise correlation between the activities of the key stress-related iModulons: Translation, ppGpp, and RpoS. The Translation and ppGpp iModulon activities are strongly positively correlated (r = 0.84), while both are negatively correlated with the RpoS iModulon activity. Coloured circles mark the 43 carbon-source experiments (Group A–D substrates). **(B) ME-model-estimated mass fractions assigned to carbon-catabolism-associated iModulon gene sets across carbon-source conditions**. Heatmap showing ME-computed mass fractions of carbon-catabolism-related iModulons (columns) across carbon source conditions (rows). For visualization, mass fractions were scaled within each iModulon to 0–1. **(C)** Stress-response landscape for the 43 carbon substrates. Heat map of activities for 24 stress-related iModulons (rows) under each carbon condition (columns). The carbon substrates are categorized into groups A-D as defined in **Figure 1**. The icon above each column denotes the central-metabolic entry route of the carbon substrate (green = glycolysis, blue = pentose-phosphate, orange = TCA-direct), and the symbol color beneath denotes the compound class (legend, bottom right). Gluc, Glucose; Glc-6-P, D-Glucose-6-phosphate; Neu5Ac, N-Acetyl-neuraminic acid; GlcNAc, N-Acetyl-D-glucosamine; GlcA, D-Glucuronic acid; GalA, D-Galacturonic acid; MePyr, Methylpyruvate; MeGal, β-Methyl-D-galactoside; L-Asp, L-Aspartic acid; α-KG, α-Ketoglutaric acid; m-Tar, m-Tartaric acid; DL-Malic, D-Malic acid; L-Pro, L-Proline; L-Asn, L-Asparagine.

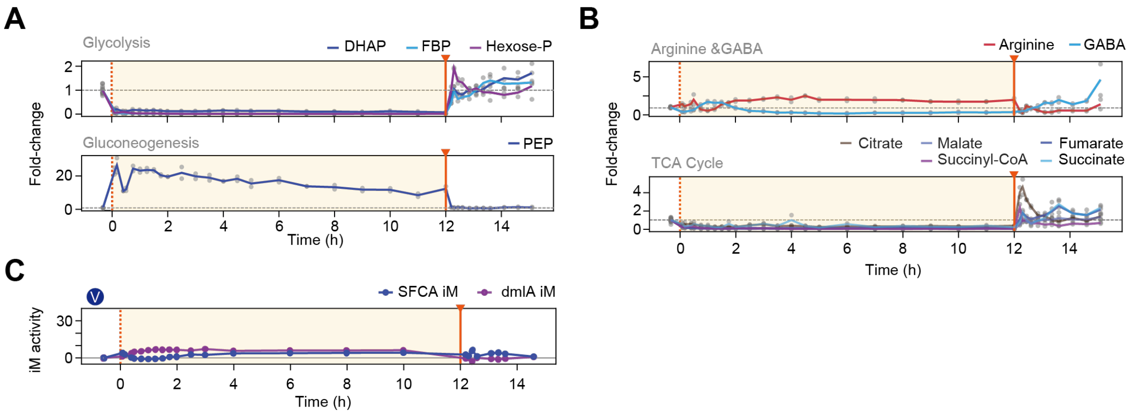

Fig. S9. Metabolic and regulatory dynamics during glucose starvation, additional data and computational results. (A) Glycolysis and gluconeogenesis metabolite dynamics. Upper panel: Glycolytic intermediates DHAP, FBP, and hexose-P showing rapid depletion upon glucose withdrawal. Lower panel: PEP accumulation indicating gluconeogenic flux. **(B)** Amino acid and TCA cycle dynamics. Upper panel: Arginine (red) shows initial accumulation from protein degradation followed by consumption; GABA (blue) remains low. Lower panel: TCA cycle intermediates (citrate, malate, fumarate, succinyl-CoA, succinate) showing coordinated depletion during starvation. **(C)** Cluster V iModulon activities remain low. SCFA iModulon (blue) and dmlA iModulon (purple) show negligible change throughout starvation, consistent with the absence of exogenous substrates associated with Cluster V activity in the single-carbon atlas. Orange-shaded regions indicate the 12-hour starvation period, and orange vertical lines mark glucose re-addition. Metabolites are shown as fold-change to the pre-starvation sample; dots show time points (replicates in gray), lines show means; beige shading denotes starvation. As in **Figure 6**, the dashed line indicates starvation onset; the orange triangle indicates glucose addition.

Legends for Datasets S1 to S3

Dataset S1 (separate file). Carbon-source growth conditions. Summary of culture conditions used for carbon-source experiments, including sample identifiers, carbon substrate and concentration, nitrogen source, base medium, categorical labels, and growth phenotype metrics (specific growth rate and z-scores relative to controls). Units are reported as provided (g/L or % w/v). The complete RNA-seq sample-level manifest is provided in Dataset S2, Metadata sheet.

Dataset S2 (separate file). Catalogue of all iModulons inferred in this study and the accompanying sample-level metadata. The iModulons sheet reports, for each ICA-derived gene module, its enriched regulator(s), enrichment statistics (p- and q-values), performance metrics (precision, recall, F1), module/regulon sizes, variance explained, and functional annotations; the Metadata sheet lists strain, condition, media, temperature, sequencing platform, and public accession identifiers for each transcriptome sample. iModulon: an independent component analysis (ICA)–derived co-regulated gene set. q-value: FDR-adjusted p-value. F1: harmonic mean of precision and recall. TP: true positives (genes overlapping the reference regulon). NA: not available.

Dataset S3 (separate file). Structural predictions for uncharacterized genes in the strongly upregulated SgcABCEQX iModulon. Comparative models were built with AlphaFold-Multimer and aligned to the closest experimentally characterized homologs using MM-align to derive global similarity scores (TM-score, RMSD) and conserved active-site features. TM-score: Template-Modelling score (0–1); values > 0.5 indicate the two proteins adopt the same fold. RMSD: root-mean-square deviation of Cα atoms after optimal superposition (Å); lower values denote closer structural overlap.
